# Natural Exponential and Three‐Dimensional Chaotic System

**DOI:** 10.1002/advs.202204269

**Published:** 2023-03-28

**Authors:** Shiwei Liu, Qiaohua Wang, Chengkang Liu, Yanhua Sun, Lingsong He

**Affiliations:** ^1^ College of Engineering Huazhong Agricultural University Wuhan 430070 China; ^2^ School of Mechanical Science and Engineering Huazhong University of Science and Technology Wuhan 430074 China

**Keywords:** chaotic system, duffing model, entropy, phase space, prediction

## Abstract

Existing chaotic system exhibits unpredictability and nonrepeatability in a deterministic nonlinear architecture, presented as a combination of definiteness and stochasticity. However, traditional two‐dimensional chaotic systems cannot provide sufficient information in the dynamic motion and usually feature low sensitivity to initial system input, which makes them computationally prohibitive in accurate time series prediction and weak periodic component detection. Here, a natural exponential and three‐dimensional chaotic system with higher sensitivity to initial system input conditions showing astonishing extensibility in time series prediction and image processing is proposed. The chaotic performance evaluated theoretically and experimentally by Poincare mapping, bifurcation diagram, phase space reconstruction, Lyapunov exponent, and correlation dimension provides a new perspective of nonlinear physical modeling and validation. The complexity, robustness, and consistency are studied by recursive and entropy analysis and comparison. The method improves the efficiency of time series prediction, nonlinear dynamics‐related problem solving and expands the potential scope of multi‐dimensional chaotic systems.

## Graphical abstract

A novel natural exponential and three‐dimensional chaotic system with higher sensitivity to initial condition is presented. Its characterizations are revealed via various chaotic dynamic indicators. The performance evaluation through recursive and entropy study verifies the model complexity and robustness, while the feasibility of this system is validated by time series prediction, image encryption, and decryption.

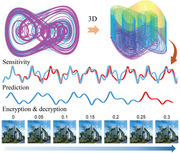

## 1 Introduction

As initiated and depicted by Lorenz through the deterministic nonperiodic flow,^[^ ^1^ ^]^ a chaotic system was first detected in the restricted three‐body problem^[^ ^1^, ^2^ ^]^ and the mazing Lorenz system with three differential equations and two quadratic terms could produce a simple attractor featuring with two aesthetic wings for the butterfly effect.^[^ ^3^ ^]^ It is usually characterized as deterministic, theoretically well organized and associated with a certain sensitivity to initial conditions, which has raised much concern in intermittently forced linear system,^[^ ^2^ ^]^ living biology,^[^ ^4^ ^]^ coupled Kerr‐nonlinear parametric oscillator,^[^ ^5^ ^]^ magnetic switching in a nanoscale ferromagnet,^[^ ^6^ ^]^ as well as metaheuristic based artificial intelligence. Although a simple form of chaotic system with some oscillators can generate complex time series, the physical mechanisms are strongly connected with the fractal properties, recurrence relation and phase space features, which are also the main factors to the dynamic motion, and source of producing an explicable strong attractor and intrinsic rules within the output numerical sequences.^[^ ^7^ ^]^ Existing two‐dimensional (2D) autonomous chaotic systems ^[^ ^5^, ^8^ ^]^ are simple with two ordinary equations coupled in the *x*–*y* plane, and the detection sensitivity for weak periodic component is usually limited by the initial condition of the chaotic system. As for three‐dimensional (3D) autonomous polynomial and even hyperchaotic systems, they are often trapped in the equilibrium state stability, where Jacobian eigenvalues are difficult to characterize, and traditional bifurcation analysis is usually unavailable in finding the chaos route.^[^ ^9^ ^]^ Therefore, three main criteria should be carefully considered in constructing an effective chaotic system: i) A well described theoretical equation and concept is required. ii) Related testing techniques should be utilized to validate the chaotic solutions and observability. iii) Real data need to be obtained to investigate or evaluate the chaotic and stochastic dynamic performance.

Aimed at the nonlinear system solution, the Runge Kutta 4th order (RK4) method, multi‐step differential transform ^[^ ^10^ ^]^ and two‐point block methods ^[^ ^11^ ^]^ are considered. Besides, the chaos edge theory^[^ ^12^ ^]^ is also proposed in resolving the Smale paradox, where complex behaviors including the static and dynamic pattern formulation emerging in the bio‐inspired array can reproduce shocking phenomenon when investigating a chaotic model from cellular biology. To overcome the chaos disappearance caused by parameter disturbance in practical applications, many new chaotic models and nonlinear systems ^[^ ^13^, ^14^ ^]^ are proposed including the transient chaos,^[^ ^15^ ^]^ spatial chaos,^[^ ^16^ ^]^ topological chaos,^[^ ^17^ ^]^ mixed multi‐chaos,^[^ ^18^ ^]^ 3D pulsed chaos.^[^ ^19^ ^]^ Although the existing exponential or logarithmic chaotic model is proposed to produce one‐dimensional (1D) chaotic maps with robust chaos,^[^ ^20^ ^]^ higher dimensional chaotic system is still needed to bring more information in understanding the dynamic motions and chaos phenomena. From the perspective of chaotic behavior performance evaluation, Poincare section,^[^ ^21^ ^]^ phase space motion,^[^ ^22^ ^]^ etc. are applied to investigate the nonlinear system sensitive dependence on initial conditions.^[^ ^23^ ^]^ Besides, quantitative tools are developed to measure the information propagation through many‐body systems in evaluating the physical mechanisms of quantum chaos.^[^ ^24^ ^]^ The compressed ultrafast photography is used in real‐time spatial‐temporal observation and imaging of an optical chaotic system,^[^ ^25^ ^]^ and the transient trajectories are considered to estimate the system parameters of Duffing oscillator,^[^ ^26^ ^]^ which all pave the way for comprehensively understanding chaotic systems. As for the chaotic system complexity, stability analysis and mutational control,^[^ ^27^ ^]^ in contrast with the traditional multiple time scale and Laplace transform,^[^ ^28^ ^]^ various entropy‐based methods^[^ ^29^, ^30^, ^31^ ^]^ are reported and proved to be effective in intrinsic law mining for a chaotic time series.^[^ ^32^ ^]^

Chaotic system and method can be applied to weak signal detection,^[^ ^33^ ^]^ security of digital image,^[^ ^34^ ^]^ climate change,^[^ ^35^ ^]^ and biomedicine monitoring,^[^ ^36^ ^]^ hardware design and optimization,^[^ ^37^, ^38^ ^]^ time series prediction^[^ ^39^ ^]^ in amplitude and frequency domain.^[^ ^40^ ^]^ Various improved and optimization methods are proposed to enhance the recognition accuracy and prediction precision from the perspective of parameter selection and adaptive adjustment, but they are limited to specific application scenarios.^[^ ^41^, ^42^ ^]^ In contrast to the Lorenz system, the Duffing model is frequently considered in the 2D autonomous system, as well as the natural exponential stochastic resonance model.^[^ ^43^ ^]^ Many attempts have been made on natural exponential functions and methods in improving the performance of traditional chaotic models applied to weak signal detection,^[^ ^33^, ^43^ ^]^ ecosystem experiment,^[^ ^44^ ^]^ quantum computation,^[^ ^45^ ^]^ astronomical observation,^[^ ^46^ ^]^ geological prediction,^[^ ^47^ ^]^ and network generation.^[^ ^48^ ^]^ For instance, the hidden attractor discovery with an exponential nonlinear term ^[^ ^49^ ^]^ shows rich dynamic behaviors, and the out‐of‐time‐ordered correlation models (OTOCs) proportional to a natural exponential function is also presented to quantify weak quantum chaos ^[^ ^50^ ^]^ and many‐body system,^[^ ^51^ ^]^ higher dimensional characterizations of a chaotic system, especially for the natural exponential function based models, are rarely studied and revealed.^[^ ^52^ ^]^ If three or higher dimensional chaotic system is expanded or coupled with 2D classical models, better performance of the chaotic system may be shown. On the other hand, although the existing 3D chaotic systems including the Lorenz and Rossler models can distinguish periodic or nonperiodic weak signals by the chaotic and dynamic motion when a slight difference is brought in these systems comparing with a standard periodic signal, the forecasting accuracy and efficiency are usually limited owing to the relative slower iteration process in the dynamic motion.

**Figure 1:**
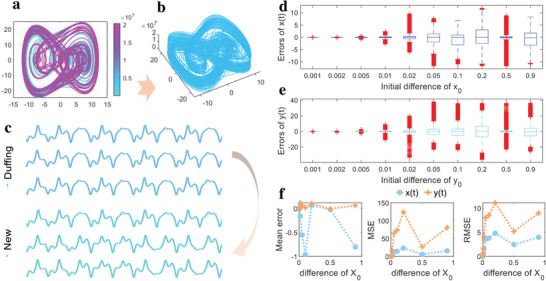
Sensitivity of natural exponential chaotic system to initial conditions. a) Plane graph of the natural exponential chaotic system attractor. b) 3D attractor of the natural exponential chaotic system. c) Output signals of *x*(*t*) under different initial conditions. The curves in the first three lines are the output results from traditional Duffing system and the lower rows with three signals are the comparison results from the natural exponential chaotic model under different initial conditions. These three curves of Duffing and the new model are all calculated with initial value (*x*(*t*), *y*(*t*), *z*(*t*)) of (1, 1, 0.1), (1.05, 1, 0.1), (1.1, 1, 0.1) from top to bottom lines. d) Error of output *x*(*t*) when the initial input difference of *x*_0_ changes. e) Error of output *y*(*t*) when *y*_0_ changes. f) Change trends of output errors for *x*(*t*) and *y*(*t*) when *x*_0_ varies with the element of [0.001, 0.002, 0.005, 0.01, 0.02, 0.05, 0.1, 0.2, 0.5, 0.9].

Considering that the natural exponential function features better signal amplification^[^ ^43^, ^53^ ^]^ and robust characterization,^[^ ^20^ ^]^ and can be widely applied to signal denoising,^[^ ^43^, ^54^ ^]^ forecast and optimization,^[^ ^55^, ^56^, ^57^ ^]^ a natural exponential and higher dimensional chaotic system is proposed and expanded referring to the classical 2D autonomous Duffing system in this work. Except for the basic *x*(*t*) and *y*(*t*) in the common 2D Duffing system, another higher dimensional term of *z*(*t*) expanded by *α* · *xe* ^*β* · *x*^is given in Equation (1).

(1) $$\left\{\begin{array}{c} \dot{x}=y\\ \dot{y}=a\cdot y+\zeta \cdot x-b\cdot x^{3}+c\cdot \cos\left(d\cdot t+g\right)\\ \dot{z}=\alpha \cdot xe^{\beta \cdot x} \end{array}\right.$$

where, *a*, *b*, *c*, *d*, *ζ*, *g*, *α*, and *β* are the main parameters of the new 3D chaotic system. Obviously, the driving force of the periodic term indicated by the cosine function remains unchanged while a natural exponential term coupled with *x*(*t*) is introduced. The 2D and 3D attractors of the natural exponential chaotic system are shown in **Figure** **1**a,b. Comparing Figure 1a with that of traditional Duffing system, we can find that the plane graph of the new chaotic system is still presented as an elliptic shape with several hyperbolic curves and homoclinic points, whereas more circular loops and segment paths can be observed both in 2D and 3D attractors, which may thus promote the chaotic and dynamic motion efficiency locally. When the main parameter of *a*, *b*, *c*, *d*, *ζ*, *g*, *α*, and *β* are set as 0.3, 0.1, 35, 1, 0, 0, 1, and 1, while the iteration time of t is set as 0.1 to 500 with a step of 0.01, the system output results of *x*(*t*) by traditional Duffing model and the natural exponential chaotic system with an initial input difference of *x*_0_ are obtained and compared in Figure 1c. It can be clearly seen that there exist relatively bigger differences in the output for the new chaotic system after several iterations under different initial conditions, while there is little difference for the common Duffing model among different output results when a slight change in the initial input conditions occurs. Namely, the new higher dimensional chaotic model is more sensitive to the initial input than that of the traditional chaotic system, which makes the new method more reliable in time series prediction and weak signal detection. Besides, when the initial input difference of *x*_0_ changes from 0.001 to 0.9, the output errors of *x*(*t*) and *y*(*t*) expressed in Figure 1f indicate that the output difference for *y*(*t*) is even larger than that of *x*(*t*). That's to say, a slight difference for the initial input of *x*(*t*) can lead to a bigger output error of *y*(*t*). As the initial difference of *x*_0_ gains and is smaller than 0.1, the mean error of *x*(*t*) decreases sharply while the mean square error (MSE) and root mean square error (RMSE) increase gradually. Meanwhile, the mean error of *y*(*t*) keeps stable and the MSE or RMSE behaves similarly to that of *x*(*t*). Furthermore, when the initial input difference of *x*_0_ and *y*_0_ changes, the box figures for the statistical errors of *x*(*t*) and *y*(*t*) expressed in Figure 1d,e manifest that when the initial differences of *x*_0_ and *y*_0_ are smaller than 0.02, the absolute errors of *x*(*t*) and *y*(*t*) are confined to a smaller scope, and there exist bigger upper and lower limits for the output errors when the differences are bigger than 0.02. In another word, the new chaotic system can not only improve the weak signal recognition resolution when a tiny initial input difference is applied, but also behaves well both in the coupled output of *x*(*t*) and *y*(*t*).

## 2 Results

### 2.1 Mapping and Bifurcation Characterization

**Figure 2:**
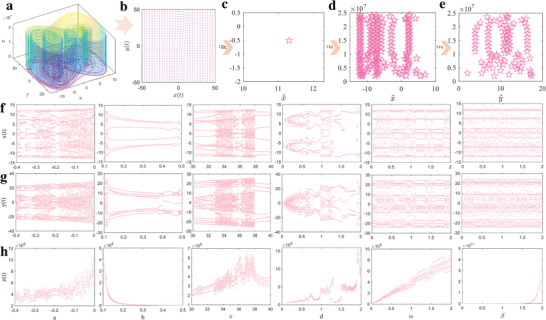
Mapping and bifurcation characterization of the natural exponential 3D chaotic system. a) 3D mapping of natural exponential chaotic system. b) Dynamic trajectory in the *x*–*y* plane. c) Poincare map in the *x*–*y* plane. d) Poincare map in the *x*–*z* plane. e) Poincare map in the *y*–*z* plane. f–h) Bifurcation map under different main system parameters (*a* = −0.4:0.001:−0.005), *b* = 0.1:0.001:0.5, *c* = 30:0.01:40, *d* = 0.005:0.01:2, *α* = 0.005:0.01:2, *β* = 0.005:0.01:2). Results for *x*(*t*), *y*(*t*), and *z*(*t*) are expressed in (f), (g), and (h), separately.

When the 3D attractor of the chaotic system is mapped in the *x*–*y* plane, the dynamic motion trajectory can be seen in **Figure** **2**a. The limit circle in the local position of the trajectory manifests that the natural exponential 3D chaotic system is stable in the *x*–*y* plane, as expressed in Figure 2b. Further Poincare section of this chaotic system projected to the *x*–*y* plane shown in Figure 2c is characterized with one point. Namely, the dynamic motion is periodic in the *x*–*y* plane, which validates the stable motion state of the limit circle. However, the chaotic characterization can also be found in the *x*–*z* and *y*–*z* plane when the Poincare section projection results exhibited in Figure 2d,e are observed, those discrete points mapped in *x*–*z* and *y*–*z* plane demonstrate that the chaotic characterizations are well kept in another two dimensions compared with traditional Duffing system, which makes the time series prediction possible.

As mentioned in Equation (1), when six main system parameters of *a*, *b*, *c*, *d*, *α*, and *β* are considered, the bifurcation diagrams for the output of *x*(*t*), *y*(*t*), and *z*(*t*) shown in Figure 2f–h indicate that the change of these parameters can discretely influence the solution stability of the natural exponential chaotic system. Explanatorily, the period doubling bifurcation for *x*(*t*), *y*(*t*), and *z*(*t*) can be seen when *a* is near the values of −0.3 and −0.2, *c* is around 32 and 36, *d* is near 1, *α* is in the neighborhood of 1 and 1.5. On the other hand, the bifurcation state of the chaotic system is unchanged when *b* and *β* vary, which demonstrates that the solution stability is insensitive to *b* and the new introduced *β*.

### 2.2 Phase Space Behavior and Performance Evaluation

**Figure 3:**
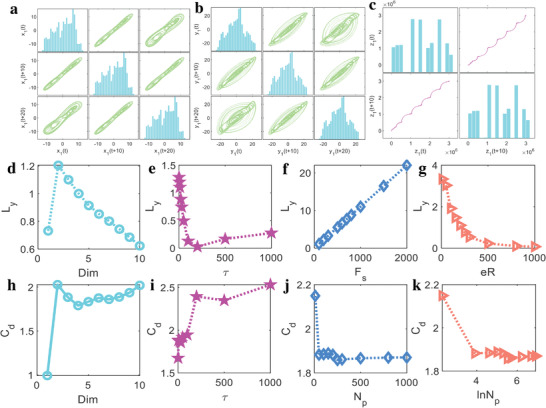
Phase space reconstructed results and performance evaluated by Lyapunov exponent and correlation dimension. a–c) Phase space reconstructed by *x*(*t*), *y*(*t*), and *z*(*t*), the diagonal plot represents the output of *x*(*t*), *y*(*t*), and *z*(*t*) under different moments. d–g) Influence of embedded dimension (Dim = [9, 10]), delay time (*τ* = [1, 5, 10, 20, 30, 50, 100, 200, 500, 1000]), sampling rate (*F*_s_ = [100, 200, 300, 500, 600, 700, 800, 1000, 1500, 2000]), and expansion range (eR = [10, 50, 100, 150, 200, 250, 300, 500, 800, 1000]) on Lyapunov exponent (*L*_y_) in the natural exponential chaotic system. h–k) Influence of Dim, *τ*, *N*_p_ (within the scope of [10, 50, 100, 150, 200, 250, 300, 500, 800, 1000]) and logarithm of *N*_p_ on *C*_d_ in the natural exponential chaotic system.

When the main parameters of *a*, *b*, *c*, *d*, *ζ*, *g*, *α*, and *β* are set as −0.3, 0.1, 35, 1, 1, 0, 1, 1, and the initial input values of *x*_0_, *y*_0_, *z*_0_ are set as 0.1, 0.1, and 1, the phase space reconstructed results for *x*(*t*), *y*(*t*), and *z*(*t*) are presented in **Figure** **3**a–c with the embedding dimension and time lag of 3 and 10. Although there exist large differences in the output of *x*(*t*), the reconstructed phase space under different time lags are all presented as periodic shapes judged by the closed and elliptical trajectories. As a contrast, the reconstructed phase space results calculated for *y*(*t*) are all mixed with additional trajectories located near the edge. As for *z*(*t*), the third dimensional phase space reconstructed trajectory is an open and homoclinic orbit. Comparing with traditional Duffing system, the flexural phase space trajectory of *z*(*t*) makes the dynamic motion of the natural exponential chaotic system more robust in the iteration process.

The relationships between four system parameters including the embedding dimension (Dim), delay time (*τ*), sampling rate (*F*_s_), range of expansion step (eR) used to estimate local expansion rate and the Lyapunov exponent (*L*_y_) for the natural exponential chaotic system are illustrated in Figure 3d–g, separately, and *L*_y_ is defined as the limit of,

(2) $$L_{y}=\lim_{t_{n}\to \infty }\frac{1}{t_{n}}\sum_{i=0}^{n-1}\ln\left|f^{\prime }\left[x\left(t_{i}\right)\right]\right|$$

where *f*’ is the 1D mapping function, *n* is the iteration number, and *x*(*t*) is the time series of the chaotic system (Supporting Information). When the system parameters are set as constant and the Lyapunov indicators change, *L*_y_ is always positive and the high dimensional system remains a chaos state. Additionally, as *τ* and eR gain from 0 to 1000 and the embedded dimension is bigger than 2, *L*_y_ is gradually decreased, which means that the chaos state weakens little by little, but *L*_y_ is increased and the chaos state become strengthened when *F*_s_ augments.

Furthermore, as an effective complexity indicator for a chaotic system attractor, the correlation dimension (*C*_d_) is described by Equation (3), *C*_n_ is the correlation integral, and *r* is the neighborhood radius between two points in the correlation integral.

(3) $$C_{d}=\log\left(C_{n}\left(r\right)\right)/\log\left(r\right)$$

where *N*_p_ is the point number to determine a good resolution for the neighborhood radius. When the chaotic system parameters are set as constants, the calculation results for *C*_d_ are expressed in Figure 3h–k. It's evident that as Dim and *τ* increase, the complexity of the attractor enhances as well, while *C*_d_ attenuates step by step when *N*_p_ or ln*N*_p_ gains. In another word, the influence of Dim and *τ* to *L*_y_ and *C*_d_ are almost the opposite, while the impact of *F*_s_, eR and *N*_p_ on the chaos state and complexity of attractors in phase space are relatively more monotonous.

**Figure 4:**
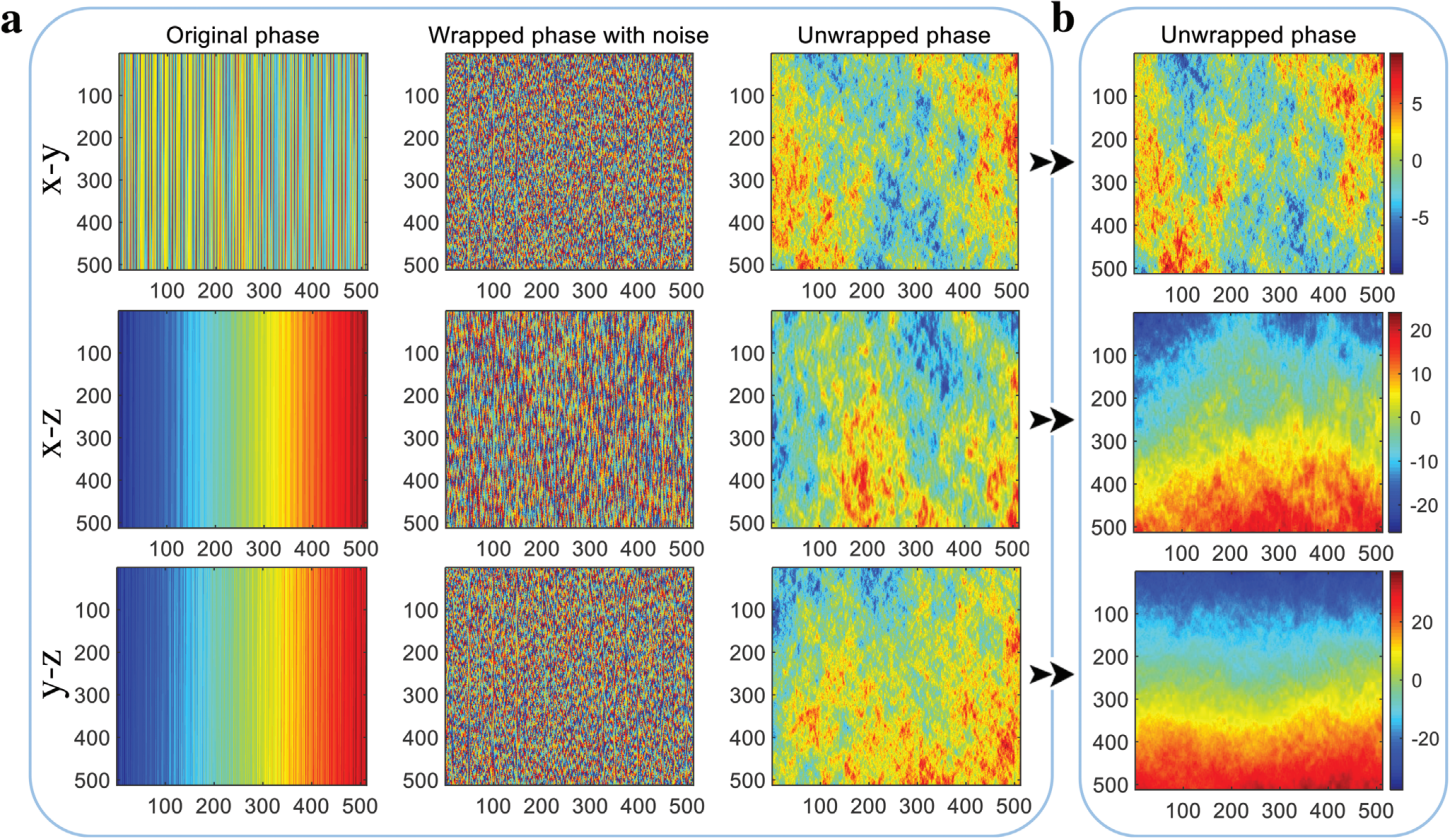
Comparison of noise induced phase. a) Noise induced phase by traditional chaotic method. b) Noise induced phase unwrapped by natural exponential and 3D chaotic system.

Noises are occasionally encountered in the time series detection and prediction, which may cause chaos in the phase of chaotic systems. As a contrast, a common Duffing system is first investigated and the system parameters are set the same as mentioned before, comparisons of the noise induced phase in three different planes are shown in **Figure** **4**a. When the 1D original output time series of *x*(*t*), *y*(*t*), and *z*(*t*) in the chaotic system are reshaped as 2D matrixes with the size of 512 × 512, the original phase can be calculated in the first column of Figure 4a, and when a random noise with an amplitude of 0.5 is wrapped with these original time series, the phase is presented as a fuzzy image, as shown in the second column of Figure 4a. When the noise induced phase is unwrapped using unweighted least‐square method,^[^ ^58^ ^]^ the results of unwrapped phase for the traditional Duffing system are presented in the right side of Figure 4a, which indicate that the original phase can still not be well distinguished after unwrapped. Namely, most of the phase information are changed or missed compared with the original phase. Nevertheless, when the new natural exponential chaotic system is applied, the unwrapped phase is exhibited in Figure 4b. It's apparent that the original phase can be well recovered through the new method with a phase shift of 90°, where the chaotic information can all be exactly extracted judged by the strength distribution. Combining these unwrapped phase results, we can preliminarily summarize that the natural exponential chaotic system has better robustness in noise induced phase change than common Duffing method.

### 2.3 Recursive and Entropy Analysis

**Figure 5:**
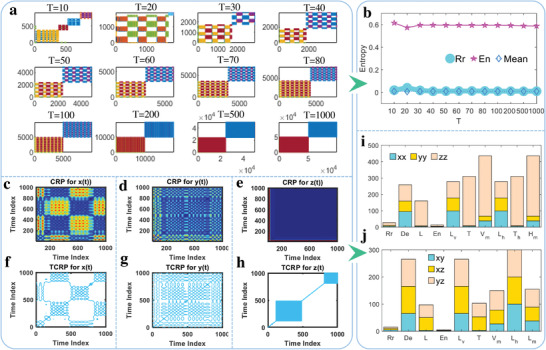
Heterogeneous and cross recurrence analysis. a) Heterogeneous recurrence results under different ending time of *T*. b) Change trends of heterogeneous recurrence rate (Rr), heterogeneous entropy (En), and heterogeneous mean (*Mean*) under different *T*. c–e) CRP results for *x*(*t*), *y*(*t*), and *z*(*t*). f–h) TCRP and synchronization lines for *x*(*t*), *y*(*t*), and *z*(*t*). i) Cross recurrence results for *x*(*t*)–*x*(*t*) (labeled as *xx*), *y*(*t*)–*y*(*t*) (labeled as *yy*) and *z*(*t*)−*z*(*t*) (labeled as *zz*). j) Cross recurrence results for *x*(*t*)−*y*(*t*) (labeled as *xy*), *x*(*t*)−*z*(*t*) (labeled as *xz*) and *y*(*t*)−*z*(*t*) (labeled as *yz*). The variables in (i) and (j) are determinism (De), entropy (En), length of longest vertical (*V*_m_) and horizontal (*H*_m_) line, length of longest (*L*) and average (*L*_v_) diagonal line, laminarity of vertical (*L*_m_) and horizontal (*L*_h_) lines, recurrence rate (Rr), trapping time of vertical lines (*T*) and horizontal lines (Th), respectively.

The heterogeneous^[^ ^59^, ^60^ ^]^ and cross recurrence analysis^[^ ^61^ ^]^ for the natural exponential chaotic system is shown in **Figure** **5**. The final time of *T* calculated for the nonlinear system is set as 10, 20, 30, 40, 50, 60, 70 and 80, 100, 200, 500, 1000, respectively. Then, the time series calculated from the natural exponential chaotic system with different lengths can be investigated from the perspective of recurrence percentage, while the heterogeneous recurrence plot of the system output presented in Figure 5a manifests that these output signals or structures of the chaotic system are equally distributed in the second group and locally uniformly spaced when *T* is bigger than 30. As *T* is set as 10, short‐term chaotic dynamic motion makes the nonlinear system presented as fractal structure segments, and continuous change of the dynamic system is frequently occurred in the localized areas. To quantify the heterogeneous recurrence characterization of the chaotic system, three main performance indicators including the heterogeneous recurrence rate of Rr, heterogeneous mean of Mean and heterogeneous entropy of En are applied, where Rr measuring the percentage of recurrence is defined as,^[^ ^59^ ^]^

(4) $$Rr={\left(\frac{{\overset{=}{W}}_{k_{1},k_{2}\cdot \cdot \cdot ,k_{L}}}{N}\right)}^{2}$$

Specifically, ${\overset{=}{W}}_{k_{1},k_{2}\text{…},k_{L}}$represents the cardinality of the state sequence set $W_{k_{1},k_{2}\text{…},k_{L}}$, *L* is the colored dots number in the heterogeneous recurrence plot, *N* is the total state number of the stochastic and nonlinear process. The heterogeneous mean of *Mean* is given by,^[^ ^59^ ^]^

(5) $$\left\{\begin{array}{c} \mathrm{Mean}=\frac{2}{\overset{=}{W}\left(\overset{=}{W}-1\right)}\sum_{m=1}^{\overset{=}{W}}\sum_{n=m+1}^{\overset{=}{W}}S_{k_{1},k_{2},\text{…},k_{L}}\left(m,n\right)\\ S_{k_{1},k_{2},\cdot \cdot \cdot ,k_{L}}\left(m,n\right)=\left\|\varphi^{m}-\varphi^{n}\right\|\;\left(\varphi^{m},\varphi^{n}\in W_{k_{1},k_{2},\cdot \cdot \cdot ,k_{L}}\right) \end{array}\right.$$

where *φ* ^m^and *φ* ^n^are the *m*th and *n*th elements of $W_{k_{1},k_{2},\text{…},k_{L}}$, and $S_{k_{1},k_{2},\text{…},k_{L}}\left(m,n\right)$ is the distance matrix. The indicator of *Mean* characterizes the average distance among these elements in the sequence set of $W_{k_{1},k_{2},\text{…},k_{L}}$, $m,n=1,2,\text{…},\overset{=}{W}$and *m* < *n*. Besides, the heterogeneous entropy of En is described as the Shannon entropy of $S_{k_{1},k_{2},\text{…},k_{L}}\left(m,n\right)$, which measures the recurrence uncertainty of a state sequence.^[^ ^59^ ^]^

(6) $$\mathrm{En}=-\sum_{q=1}^{Q}p\left(q\right)\ln p\left(q\right)\begin{array}{cc} & \left(q=1,2,\cdot \cdot \cdot ,Q\right) \end{array}$$

where the probability distribution of $S_{k_{1},k_{2},\text{…},k_{L}}\left(m,n\right)$is given by Equation (7), *Q* is the equal bins number of $S_{k_{1},k_{2},\text{…},k_{L}}\left(m,n\right)$divided from 0 to the maximum value, and *q* = 1, 2, …, *Q*.

(7) $$\begin{array}{c} p\left(q\right)=p\left\{\;\frac{1}{\overset{=}{W}\left(\overset{=}{W}-1\right)}|\frac{q-1}{Q}\max\left(S\right)<S_{k_{1},k_{2},\text{…},k_{L}}\left(m,n\right)\leq \frac{q}{Q}\max\left(S\right)\;\right\} \end{array}$$

The quantitative heterogeneous recurrence analysis results presented in Figure 5b manifest that Rr is consistent with that of the heterogeneous mean, and the heterogeneous entropy of En also remains unchanged as *T* increases from 10 to 1000. Namely, the recurrence rate and average distance of a state sequence for the new chaotic system is very robust and stable in the whole time‐varying process, which may contribute to quantitative chaotic recognition compared with other systems revealed through qualitative strategies. On the other hand, the probability distribution and uncertainty of the state sequence is also identical, which means that the natural exponential chaotic system has strong robustness and stable model structure. As a complementary recurrence analysis strategy, the cross recurrence plot (CRP) is also calculated and shown in Figure 5c–j. The CRP, threshold cross recurrence plot (TCRP) and synchronization line expressed in Figure 5c,d and f,g for the output of *x*(*t*) and *y*(*t*) demonstrate that these time series are equally distributed, and obvious diagonal lines can be found in Figure 5f,g. Namely, there exists good consistency for these time series, but the CRP of *y*(*t*) is more refined than *x*(*t*). As for the CRP of *z*(*t*), the computational results shown in Figure 5e,h indicate that these time series are nearly constants, which coincides with the chaotic system structure and model analysis mentioned in Figure 3c. Besides, different groups of quantitative CRP analysis evaluated by De, En, *H*_m_, *L*, *L*_h_, *L*_v_, *L*_m_, Rr, *T*, *T*_h_, *V*_m_ are expressed in Figure 5i,j where 10 and 9 main indicators are separately compared. Although the entropy of En and recurrence rate of Rr are smaller than other eight indicators, almost all these ten performance indicators shown in Figure 5i for *z*(*t*) are relatively bigger than that of *x*(*t*) and *y*(*t*), which manifests that the third dimension of the natural exponential chaotic system have been expanded and the chaos motion can be successfully amplified compared with the common 2D duffing or other nonlinear chaotic systems. These quantitative TCRP indicators expressed in Figure 5j further prove this conclusion. Explanatorily, the cross recurrence calculated for *x*(*t*)−*z*(*t*) and *y*(*t*)−*z*(*t*) are more evenly distributed than that of the self‐cross recurrence of *x*(*t*)−*x*(*t*) and *y*(*t*)−*y*(*t*), especially for *V*_m_, which suggests that the new model with additional term of *z*(*t*) is more stable and robust than other 2D chaotic systems.

The topological entropy (TE) is defined as the number of orbit points with a length of *n*, such as,

(8) $$TE=\lim_{n\to \infty }\ln\frac{N(n)}{n}$$

**Figure 6:**
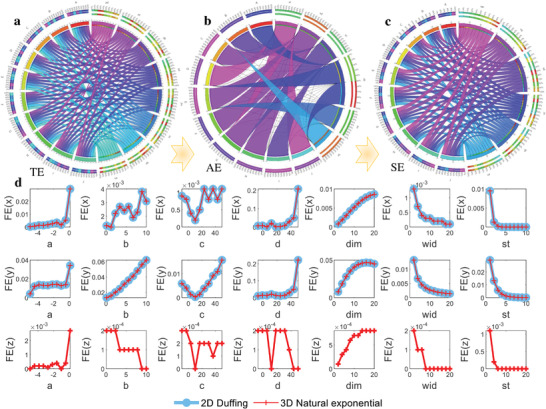
Entropy analysis and comparison of the chaotic systems. a) Chordal graph for topological entropy (TE). b) Chordal graph for approximate entropy (AE). *xL* and *xr* represent the AE computation results of *x*(*t*) when the parameters of *L* and r vary within the scope of [2, 4, 6, 8, 10, 12, 14, 16, 18, 20] and [0.01, 0.1, 0.2, 0.3, 0.4, 0.5, 0.6, 0.7, 0.8, 0.9]. c) Chordal graph for Shannon entropy (SE). *xq* and *xw* mean the SE computation results of *x*(*t*) when the parameters of *q* and *w* varies within the scope of [2, 4, 6, 8, 10, 12, 14, 16, 18, 20] and [1, 2, 3, 4, 5, 6, 7, 8, 9, 10]. *xa*, *xb*, *xc*, and *xd* in (a)–(c) express the corresponding entropy of *x*(*t*) when the system parameters of *a*, *b*, *c*, and *d* varies within the scope of [−5, −1, −0.5, −0.3, −0.2, −0.1, −0.01, 0, 0.01, 0.1], [0.1, 0.2, 0.3, 0.5, 1, 2, 3, 5, 7, 10], [−10, −5, −1, 0, 1, 5, 10, 20, 30, 50], and [−7, −5, −1, 0, 1, 3, 5, 15, 20, 50], respectively. A–J represent different groups of system parameters. d) Fuzzy entropy of *x*(*t*) (upper row, FE(*x*)), *y*(*t*) (middle row, FE(*y*)), and *z*(*t*) (lower row, FE(*z*)), where dim = [2, 4, 6, 8, 10, 12, 14, 16, 18, 20], wid = [2, 4, 6, 8, 10, 12, 14, 16, 18, 20], and st = [1, 2, 3, 4, 5, 6, 7, 8, 9, 10].

Accordingly, when ten groups of main chaotic system parameters of *a*, *b*, *c*, *d* are changed, the TE computed for the output of *x*(*t*) can be expressed with a chordal graph shown in **Figure** **6**a, As these parameters of *a*, *b*, *c*, *d* vary, TE is always evenly distributed and near the absolute value of 0.93. Videlicet, the topological dynamic system of the nonlinear model is simple and steady. Furthermore, the approximate entropy (AE) describing the regularity of a system is given by,^[^ ^62^ ^]^

(9) $$AE=\Phi^{m}\left(r\right)-\Phi^{m+1}\left(r\right)$$

where the variable of Φ^m^(*r*) and $C_{i}^{m}\left(r\right)$is defined as Equation (10),^[^ ^62^ ^]^ and *u*(*i* + *k*−1) is the element of *x*(*t*).

(10) $$\begin{array}{c} \left\{\begin{array}{c} \Phi^{m}\left(r\right)={\left(N-m+1\right)}^{-1}\sum_{i=1}^{N-m+1}\log\left(C_{i}^{m}\left(r\right)\right)\\ C_{i}^{m}\left(r\right)=\left(j,\max_{k=1,2,\cdot \cdot \cdot ,m}\left(\left|u\left(i+k-1\right)-u\left(j+k-1\right)\right|\right)\leq r\right)/\\ \;\;\left(N-m+1\right) \end{array}\right. \end{array}$$

As shown in Figure 6b, except for the main system parameters of *a*, *b*, *c*, and *d*, additional influence factors of window length *L* and similarity measure *r* in AE are also compared. Different from TE, there exist discrete strong and weak connectivity between these parameters and AE calculated results for *x*(*t*), which are exhibited as connected areas and lines, respectively. For instance, there exists strong coupling relationship between *xa* and *E*. When *a* is set as −0.2, the AE for *x*(*t*) is relatively bigger and the corresponding chaotic system is more irregular than others. Similarly, the connectivity between *xc* and *C* is weaker than other situations. As a measuring indicator for the information uncertainty, Shannon entropy (SE) is usually defined by,

(11) $$SE=-k\cdot \sum_{i=1}^{N}p_{i}\ln p_{i}$$

where *k* is a constant of 1, *p*_i_ is the probability of the *i*th state of the chaotic system. As shown in Figure 6c, except for four main chaotic system parameters of *a* to *d*, the embedding dimension of *w* and the uniform intervals number of *q* used in the quantification of the time series are also investigated for SE. Although the SE for *xq* and *A* is relatively smaller than others, SE for *x*(*t*) remains uniformly distributed and evenly connected when these ten groups of parameters change in most cases. The information uncertainty of the natural exponential chaotic system is immune to these changing parameters.

As mentioned in Equation (10), the fuzzy entropy (FE) measuring the complexity and probability of the new generating dynamic mode for a system is given by,^[^ ^63^ ^]^

(12) $$FE=\lim_{N\to \infty }\left[\ln\left(\Phi^{m}\left(r\right)\right)-\ln\left(\Phi^{m+1}\left(r\right)\right)\right]$$

Likewise, three additional parameters including the embedded dimension (dim), the width (wid), and step (st) of the fuzzy exponential function are also considered. When these seven groups of parameters change, FE calculation results for *x*(*t*), *y*(*t*) in the common Duffing system and *z*(*t*) in the new 3D chaotic system are obtained in Figure 6d. Obviously, FE(*x*) and FE(*y*) are always consistent for both the two comparing chaotic systems by observing the coincident curves. There exist positively related relationships between FE(*x*) and *a*, *d*, dim, as well as FE(*y*) and *a*, *b*, *d*, dim, while they are negatively related between FE and wid, st. In short, the complexity of the natural exponential chaotic system is sensitive to the change of these seven parameters, but additional *z*(*t*) in the new chaotic system will make it more robust for complexed information.

**Figure 7:**
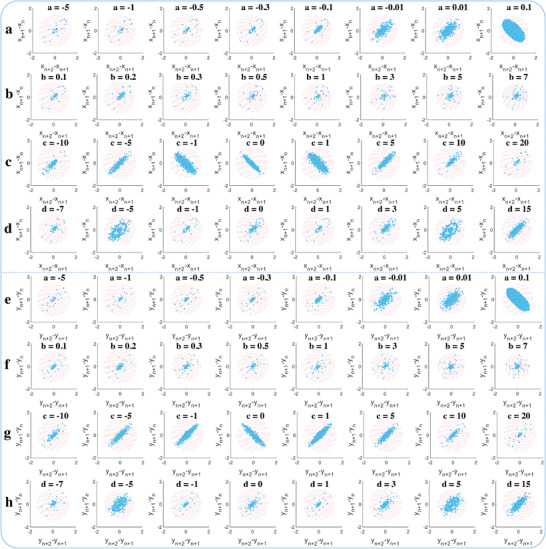
Time series entropy (TSE) measurement for *x*(*t*) and *y*(*t*). a–d) TSE results for *x*(*t*) when the system parameter of *a*, *b*, *c*, and *d* changes. e–h) TSE for *y*(*t*) when the parameter of *a*, *b*, *c*, and *d* changes. These parameters are set as *a* = [−5, −1, −0.5, −0.3, −0.1, −0.01, 0.01, 0.1], *b* = [0.1, 0.2, 0.3, 0.5, 1, 3, 5, 7], *c* = [−10, −5, −1, 0, 1, 5, 10, 20], *d* = [−7, −5, −1, 0, 1, 3, 5, 15]. The red line represents the number of circles in the circled TSE measurement, and the blue dots express 15‐degree of difference plot, which calculate these data fall over the circled‐pieced area.

The new natural exponential chaotic system is further studied from the perspective of time series entropy analysis in **Figure** **7**.

When the chaotic system parameter of *a* as mentioned in Equation (1) increases from −5 to 0.1, the time series entropy (TSE) ^[^ ^64^ ^]^ for *x*(*t*) and *y*(*t*) shown in Figure 7a,e become more and more collective around the circle center region. The more the parameter of *a* increases, the better TSE convergences and the bigger output diversity *x*(*t*) and *y*(*t*) produce. As the system parameter of *b* changes from 0.1 to 7, the TSE results of *x*(*t*) and *y*(*t*) exhibited in Figure 7b,f are discretely distributed, which manifests that the diversity or convergency of the chaotic system is slightly influenced by the parameter of *b*. Observed by Figure 7c,g, the change of parameter *c* makes the output of *x*(*t*) and *y*(*t*) unidirectionally convergent, especially within the scope of [−5, 5], but when *c* is 0, the new chaotic system without driving force is featured with an opposite convergent direction. As for the periodic term in the chaotic system, *d* can make a difference to the circled TSE of *x*(*t*) and *y*(*t*) in localized scope when *d* = −5 or *d* ≥ 5. The natural exponential chaotic system is more convergent than others, which can be seen from Figure 7d,h. Besides, the TSE of *x*(*t*) and *y*(*t*) resemble to each other regarding the sensitivity to these four main changing parameters.

**Figure 8:**
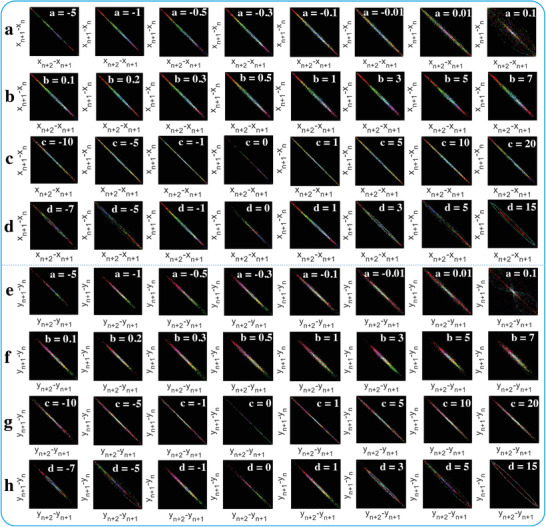
Grid entropy (GE) measurement for *x*(*t*) and *y*(*t*). a–d) GE for *x*(*t*) when the system parameter of *a*, *b*, *c*, and *d* changes as mentioned in Figure 7. e–h) GE for *y*(*t*) when the parameter of *a*, *b*, *c*, and *d* changes as mentioned in Figure 7. The range shown in each box is *x*∈[−500, 500] and *y*∈[−500, 500].

To further extract elaborated time series information of the chaotic system output, grid entropy (GE) analysis for *x*(*t*) and *y*(*t*) are presented in **Figure** **8**. When the chaotic system parameter of *a* increases from −5 to 0.1 as mentioned before in Figure 7, almost all the time series data can fall into the diagonal grid region, and similar GE distribution can be found in Figure 8b–d when *b*, *c*, and *d* varies. Besides, the GE for *x*(*t*) and *y*(*t*) are similar both in distribution and changing trend as these parameters vary. However, several discrete points are appeared in the last group when *a* is 0.1, and an annular shape composed of these discrete grid points can also be found when *d* is 15, as presented in Figure 8a,d and Figure 8e,h. These GE results provided and analyzed above demonstrate that the data convergence and aggregation of the system output cannot be easily affected by changing these chaotic system parameters, and the new natural exponential chaotic system features strong robustness and consistency.

### 2.4 Application in Prediction and Encryption

**Figure 9:**
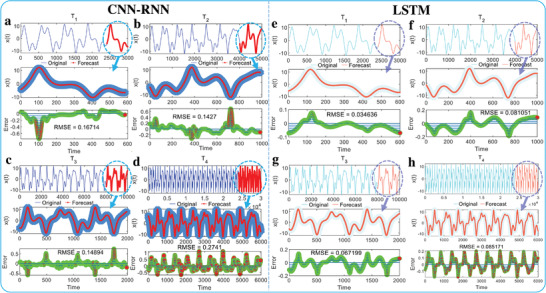
Application of the natural exponential chaotic system in time series prediction. a–d) Time series prediction results of *x*(*t*) through convolutional and recurrent combined neural network (CNN‐RNN) with time series lengths of 3000, 5000, 10 000, and 30 000. The root mean square error (RMSE) for the predicted time series of *T*_1_ to *T*_4_ is 0.1671, 0.1427, 0.1489, and 0.2741, respectively. e–h) Time series prediction results of *x*(*t*) by long‐short term memory neural network (LSTM) with time series lengths of 3000, 5000, 10 000, and 30 000. The RMSE for the predicted *T*_1_ to *T*_4_ is 0.03464, 0.08105, 0.06720, and 0.08517, respectively. The first line box shows the original signals and the forecast new signal, the second line box is a local show for the forecast new signal and corresponding original signal segments, and the third line box represents the error between the original signal and forecast new signal.

The new natural exponential chaotic system is also predictable in time domain. Primarily, prevalent deep learning networks are considered to validate the time series prediction performance. When the convolutional and recurrent combined neural network (CNN‐RNN)^[^ ^65^, ^66^ ^]^ is built, where the output data of *x*(*t*) calculated from the new natural exponential chaotic model are first split into training and testing dataset, while 80% of these data are fed as training and 20% for testing set. The learning rate is set as 0.005, the solver is adam and the maximum epoch is 250. Besides, the output of *x*(*t*) obtained under different final times including 30 s (*T*_1_), 50 s (*T*_2_), 100 s (*T*_3_), and 300 s (*T*_4_) is separately investigated. Thereafter, these time series signal datasets with lengths of 3000, 5000, 10 000, and 30 000 are presented, such as the result shown in **Figure** **9**a,d. Although the lengths of these time series are different, 80% of each dataset is applied for training and the left 20% for forecasting. Obviously, the original signals of *x*(*t*) and the forecasted 20% segments in the end coincide well with each other observed from the second line for each result in Figure 9a–d. The quantitative absolute error curves and RMSE calculation results in the third line box both demonstrate the predictability and feasibility of application in time series forecast. As a comparison, when the same datasets of *T*_1_ to *T*_4_ are applied in the time series prediction through long short‐term memory (LSTM)^[^ ^67^, ^68^ ^]^ neural network, the forecast results are shown in Figure 9e–h. Similarly, when these datasets are split into 80% for training and the left for testing, and a common LSTM architecture is built with an initial learning rate of 0.005, gradient threshold of 1, maximum epoch of 250, 20% original signals can be well tested and exactly predicted by LSTM for all the *T*_1_ to *T*_4_. Furthermore, the absolute errors predicted by LSTM are much smaller than that of the CNN‐RNN method for each of these datasets, as well as RMSE. In a word, the time series of the new natural exponential chaotic system can be predicted and is promising in extensively application fields of meteorology, biology, geology, agriculture, and mechanics.

**Figure 10:**
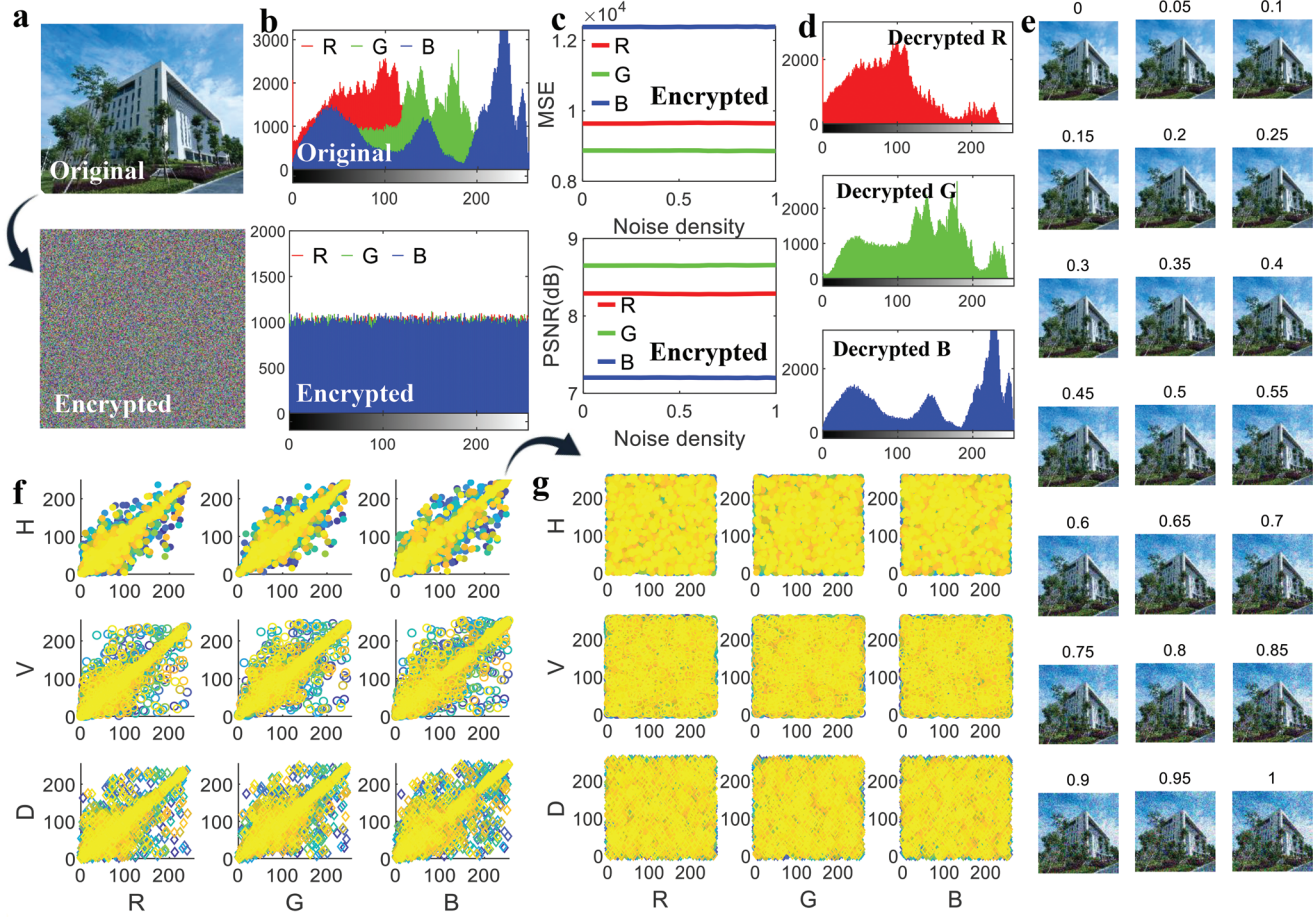
Application of the natural exponential chaotic system in image encryption and decryption. a) Original image before and after encrypted. b) RGB values of the image before and after encrypted. c) Mean square error (MSE) and peak signal‐to‐noise ratio (PSNR) of the encrypted image under different noise density. d) RGB values of the decrypted image. e) Decrypted image under different noise density. f) Correlation analysis for the original image in three dimensions. g) Correlation analysis for the encrypted image in three dimensions. *H*, *V*, and *D* in (f) and (g) means the horizontal, vertical, and diagonal correlation results, separately. The labels marked in (e) represent the Gaussian noise intensity added to the original image before decrypted.

The new chaotic system can also be applied in image encryption and decryption, as shown in **Figure** **10**. The original image is first decomposed into RGB channels, and the output time series solved by the chaotic system are applied in the DNA computation to determine the coding rule, then the original image is partitioned by DNA operation and the encrypted through RGB channel merging. The detailed RGB histograms for the original and encrypted images shown in Figure 10b indicate that the original RGB can be distinguished clearly while the encrypted RGB histograms are featured with stochastic noises. Further MSE and peak SNR (PSNR) for the encrypted image shown in Figure 10c manifest that the MSE decreases sharply as the salt and pepper noise density is bigger than 0.3, while the PSNR of all the three RGB channels keeps consistent. Similarly, when the image decryption is applied using the inverse process of DNA coding and chaotic decryption, the RGB channels of the original image can be well restored and extracted, as shown in Figure 10d. Furthermore, when the original images mixed with Gaussian noise density from 0 to 1 with an increasing step of 0.05, the image decryption results presented in Figure 10e demonstrate that the natural exponential chaotic model‐based image decryption method is also feasible and applicable. Additionally, the correlation analysis for the original image in horizontal, vertical and diagonal directions shown in Figure 10f,g indicates that there exist strong correlation relationships between neighboring pixels in the original image, while there scarcely exists correlation in all the three directions for the encrypted image, which means that the chaotic system‐based image encrypted method is valid.

## 3 Discussion

First, the natural exponential 3D chaotic system is built to improve the performance of traditional 2D autonomous Duffing system, where a natural exponential function is mainly considered in the third dimension, and a group of system parameters are introduced to adjust the chaos state and dynamic performance. When an initial difference is considered in the system input, the output results demonstrate that the new system is more sensitive to the initial input values. Further investigation about the influence of these input difference on the output time series manifests that both the 2D output of *x*(*t*) and *y*(*t*) possess higher sensitivity to the tiny disturbance in the system input with smaller errors than common Duffing system. Different with the 2D chaotic system, the new 3D nonlinear system can not only keep the chaos and periodic dynamic motion characterizations in 2D plane, but also make the third dimension fast to reach the steady state.

Later, the Poincare mapping and bifurcation characterizations are investigated. Different with common chaotic systems, although the limit circle in *x*–*y* plane is presented as a stable state, the Poincare projection in *y* and *z* plane are chaotic. The bifurcations are interruptedly appeared when these main system parameters change, which provides a reference to the best system parameter selection when a stable state is needed. Besides, these Poincare mapping and bifurcation analysis suggests that more chaotic information can be found in the dynamic motion when higher dimensional characterizations are revealed.

Then, the performance of the new chaotic system evaluated from the perspective of phase space reconstruction, unwrapping, Lyapunov exponent and correlation dimension analysis prove its robustness and changing trends. In most cases, the chaotic moving state cannot be well described from 1D time series, phase space and recurrence analysis for *x*(*t*), *y*(*t*), and *z*(*t*) provide us a clearer way to understand the evolution process and inherent law for each of the dimension. Besides, suitable selection of the embedded dimension, delay time and other parameters is significant in determining a chaotic system, especially when the nonlinear model is expanded to a hyperchaotic state.

Last, various entropy calculation and analysis results reveal the complexity, stability, aggregation, and similarity of the natural exponential chaotic system, as well as the entropy relationships between different dimensional elements. Although the influence of these model parameters to the entropy is usually monotonic, they reflect the system sensitivity and changeability. Noteworthily, the chaos state is always consistent when these main chaotic system parameters changes, indicates that the model parameters are robust in building the 3D chaotic system.

As for the limitations, we only consider the single periodic driving forces as a cosine function without any induced noise. Actually, various types of noises are also important to the stochastic resonance of the traditional Duffing system. Furthermore, other forms of natural exponential functions in composing of the third dimension of *z*(*t*) are also waited to be explored in the future work, especially for the physical mechanism of these higher dimensional and even hyperchaotic systems. Nevertheless, this chaotic system can not only be widely applied in the fields of meteorology, biology, geology, agriculture, traffic, and mechanics, but also provide a new view in understanding the complex system and studying the nonlinear phenomena.

## 4 Experimental Section

### Poincare Section and Bifurcation Diagram

Poincare section is defined as the intersection point between phase trajectory and Poincare plane of a chaotic system, and the Poincare map composed of discrete projection points is used to investigate the dynamic characterizations of the nonlinear system in the multi‐dimensional space. Typically, the dynamic motion can be judged as periodic when only one point or a few of discrete points are observed in the map, while it's quasi periodic motion if these discrete points are shaped as a closed curve. Otherwise, these motions are chaotic assuming that these mapping points are densely and randomly distributed.

As an effective indicator for characterizing the stability of the chaotic system structures and nonlinear solutions, combining the chaotic system mentioned in Equation (1), bifurcation diagram is used to study the influence of these main model parameters of *a*, *b*, *c*, *d*, *α*, and *β* on the stability of the dynamic motion. If the topological structure is changed when any minor disturbance caused by the variation of the parameter is occurred, a bifurcation in the diagram will appear. When one of the main parameter changes, other system parameters are set as *a* = −0.3, *b* = 0.1, *c* = 35, *d* = 1, *α* = 1, *β* = 1, and *g* = 0. The initial value of *x*(*t*), *y*(*t*), and *z*(*t*) are 0.1, 0.1, and 1, the duration of the computation is 100 s.

### Phase Space Unwrapping

The 2D phase wrapping and least‐square phase unwrapping methods are used to validate the sensitivity of the chaotic system to a random noise. The relationships between the wrapped and unwrapped phases can be described as,

(13) $$\varphi_{i+1,j}+\varphi_{i-1,j}+\varphi_{i,j+1}+\varphi_{i,j-1}-4\varphi_{i,j}=\Delta_{i,j}^{I}-\Delta_{i-1,j}^{I}-\Delta_{i,j}^{J}-\Delta_{i,j-1}^{J}$$

where $\Delta_{i,j}^{I}$ and $\Delta_{i,j}^{J}$ represent the wrapped phase difference in the *i* and *j* indices. Equation (13) can be simplified as$\rho_{i,j}=\Delta_{i,j}^{I}-\Delta_{i-1,j}^{I}-\Delta_{i,j}^{J}-\Delta_{i,j-1}^{J}$, *m* and *n* are the differences of *i* and *j*, which satisfy *i* = 0, 1, …, *M*−2, *j* = 0, 1, …, *N*−1. Then, discrete cosine transform is conducted to obtained *ρ*_i,j_, and the unwrapped phase can be calculated through inverse discrete cosine transform as follows,^[^ ^58^ ^]^

(14) $${\hat{\varphi }}_{i,j}=\frac{{\hat{\rho }}_{i,j}}{2\left(\cos\frac{\pi i}{M}+\cos\frac{\pi j}{N}-2\right)}$$

### Deep Learning Architecture of CNN‐RNN

The deep learning based conventional neural network (CNN) and recurrent neural network (RNN) are mainly applied in the chaotic time series forecast. These original datasets are first obtained through setting different end time, then they will be normalized as standard data through standard deviation method and split into training (80%) and testing (20%) parts. The CNN using AlexNet architecture extracts the main features of the chaotic time series, while the RNN is applied to predict the next value. After the datasets are input in the network, five conventional layers with size of 1 × 32 and an average pooling layer are used, where the excitation function of ReLu is considered. Then the CNN is finished by unfolding and flatting, while the following connected RNN composed of two long short‐term memory (LSTM) layers with length of 1 × 128 and 1 × 32 is designed. Finally, the predicted time series can be obtained through a full connected layer and output layers.

### Image Encryption and Decryption

To validate the feasibility of the proposed natural exponential chaotic system, the original image is decomposed into three 2D matrixes of *R*, *G*, and *B* channels first, and these matrixes of *M*(*R*), *M*(*g*), and *M*(*B*) are then divided into blocks with same size, where the blank elements are compensated as zeros. When the time series of the natural exponential chaotic system are generated, they will be reshaped as a matrix with the size of *M* × *N*, and these numerical elements are normalized within the scope of [0, 255]. Then, a four‐dimensional hyperchaotic time series of *X*, *Y*, *Z*, and *H* can be obtained through Runge Kutta solver and Bit AND operation, the initial values are calculated as,^[^ ^69^ ^]^

(15) $$\left\{\begin{array}{c} X(0)=\mathrm{sum}(\mathrm{sum}(\mathrm{BitAND}(M(R),17)))/(17\cdot M\times N)\\ Y(0)=\mathrm{sum}(\mathrm{sum}(\mathrm{BitAND}(M(G),34)))/(34\cdot M\times N)\\ Z(0)=\mathrm{sum}(\mathrm{sum}(\mathrm{BitAND}(M(R),68)))/(68\cdot M\times N)\\ H(0)=\mathrm{sum}(\mathrm{sum}(\mathrm{BitAND}(M(B),136)))/(136\cdot M\times N) \end{array}\right.$$

As for the *R*, *G*, and *B* values for the original image, combining the modular arithmetic (Mod) and round off operation, it can be encoded with the chaotic time series as,^[^ ^69^ ^]^

(16) $$\left\{\begin{array}{c} Q=M\mathrm{od}\left(\mathrm{round}\left(Q\times 10^{4}\right),8\right)+1\;\left(Q=X,Y,H\right)\\ Z=M\mathrm{od}(\mathrm{round}\left(Z\times 10^{4}\right),4)+1 \end{array}\right.$$

There are 8 DNA encoding forms for *X*, *Y*, *H*, and 4 operations including the plus, minus, XOR and XNOR for *Z*. Thereafter, each of these blocks for *R*, *G*, *B* channels are merged, and a diagonal traversing method is applied to scramble the image. Finally, the encrypted image can be obtained after the *R*, *G*, *B* channels are combined. Additionally, the chaotic and DNA encoded image can be decrypted if the reverse processes mentioned above are applied.

### Hardware and Software Configuration

The numerical results are mainly calculated through MATLAB R2019a, Python 3.7.9 on a 64‐bit Windows 10 operating system, 16.0 GB RAM, Intel(R) Core (TM) and i7‐7700HQ CPU @ 2.80 GHz. The calculating errors are slightly existed by repeated trials, which depends on the precise of the software and hardware.

## Conflict of Interest

The authors declare no conflict of interest.

## Supporting information

Supporting Information

## Data Availability

The data that support the findings of this study are available from the corresponding author upon reasonable request.
