# Supplementary material for: Natural Exponential and Three‐Dimensional Chaotic System
Source: Adv Sci (Weinh). 2023 Mar 28;10(15):2204269. doi: 10.1002/advs.202204269 (PMC10214267; doi:10.1002/advs.202204269)
Supplement: Supplementary file 1 — Supporting Information [file ADVS-10-2204269-s001.pdf]

ADVANCED SCIENCE
Open Access

Supporting Information

for *Adv. Sci.*, DOI 10.1002/advs.202204269

Natural Exponential and Three-Dimensional Chaotic System

Shiwei Liu, Qiaohua Wang, Chengkang Liu, Yanhua Sun and Lingsong He*

Supplementary Materials for

Natural exponential and three-dimensional chaotic system

Shiwei Liu[1,†], Qiaohua Wang[1], Chengkang Liu[1], Yanhua Sun[2], Lingsong He[2]

[1]*College of Engineering, Huazhong Agricultural University, Wuhan, 430070, China.*
[2]*School of Mechanical Science and Engineering, Huazhong University of Science and Technology, Wuhan, 430074, China.*

Supplementary Note 1. Forecasting Verification

Table S1. Influence of initial difference of x(t) to system output error.

Initial difference of x0	0.001	0.002	0.005	0.01	0.02	0.05	0.1	0.2	0.5	0.9
Mean error	-0.0012	-0.0023	-0.0129	0.0315	-0.1599	-0.5516	-0.9600	0.0675	-0.0202	-0.8016
MSE	0.0003	0.0014	0.0357	0.4069	2.5140	12.7870	14.3257	23.1781	6.0149	15.8406
RMSE	0.0182	0.0380	0.1890	0.6379	1.5856	3.5759	3.7849	4.8144	2.4525	3.9800

Table S2. Influence of initial difference of y(t) to system output error.

Initial difference of y0	0.001	0.002	0.005	0.01	0.02	0.05	0.1	0.2	0.5	0.9
Mean error	-0.0000	-0.0001	-0.0003	0.0132	0.1202	0.0703	0.0181	0.1083	-0.0069	0.0688
MSE	0.0030	0.0127	0.2928	2.7679	16.8700	64.9222	73.5096	123.7480	26.7623	80.9466
RMSE	0.0549	0.1128	0.5411	1.6637	4.1073	8.0574	8.5738	11.1242	5.1732	8.9970

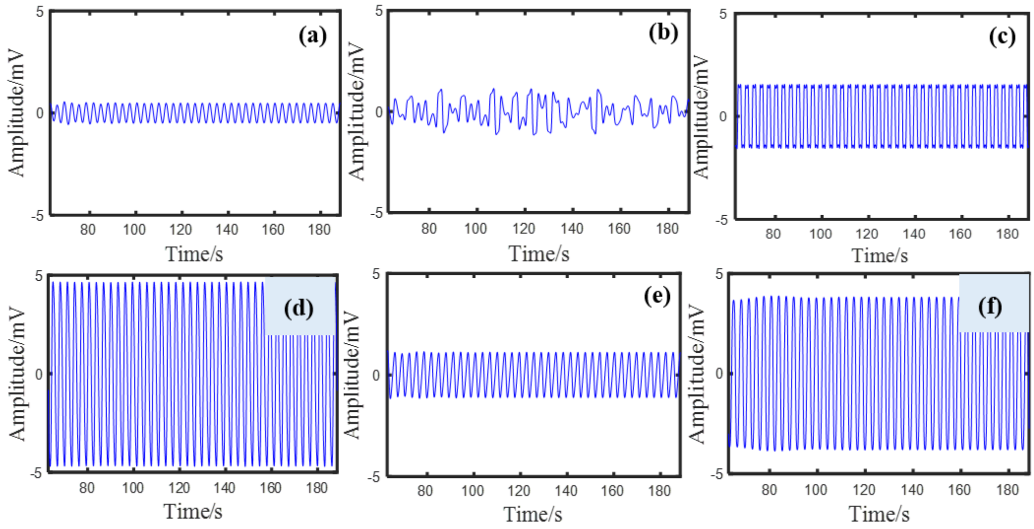

† Corresponding author, E-mail: hustliusw@sina.com

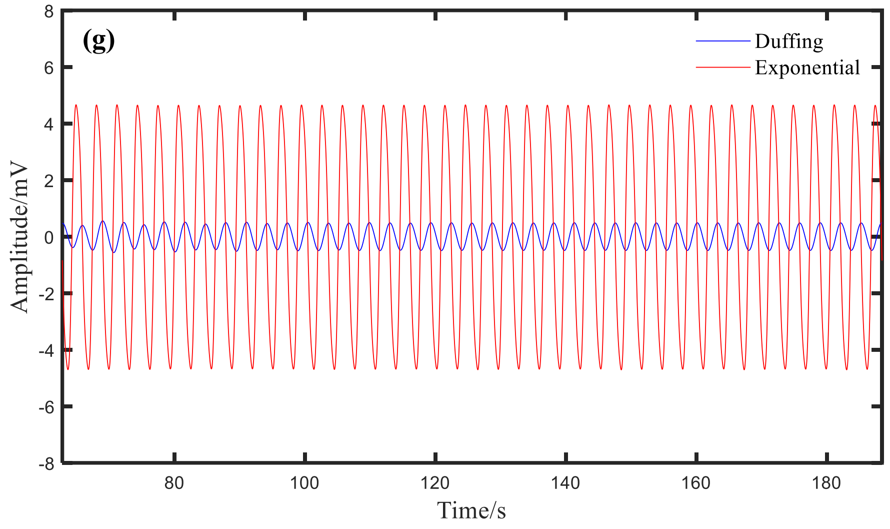

Figure S1. Output signals simulated from different SR models. (a) Output signal from M1; (b) M2; (c) M3; (d) M4; (e) M5; (f) M6; (g) Comparison of natural exponential and duffing model in output signal amplitude.

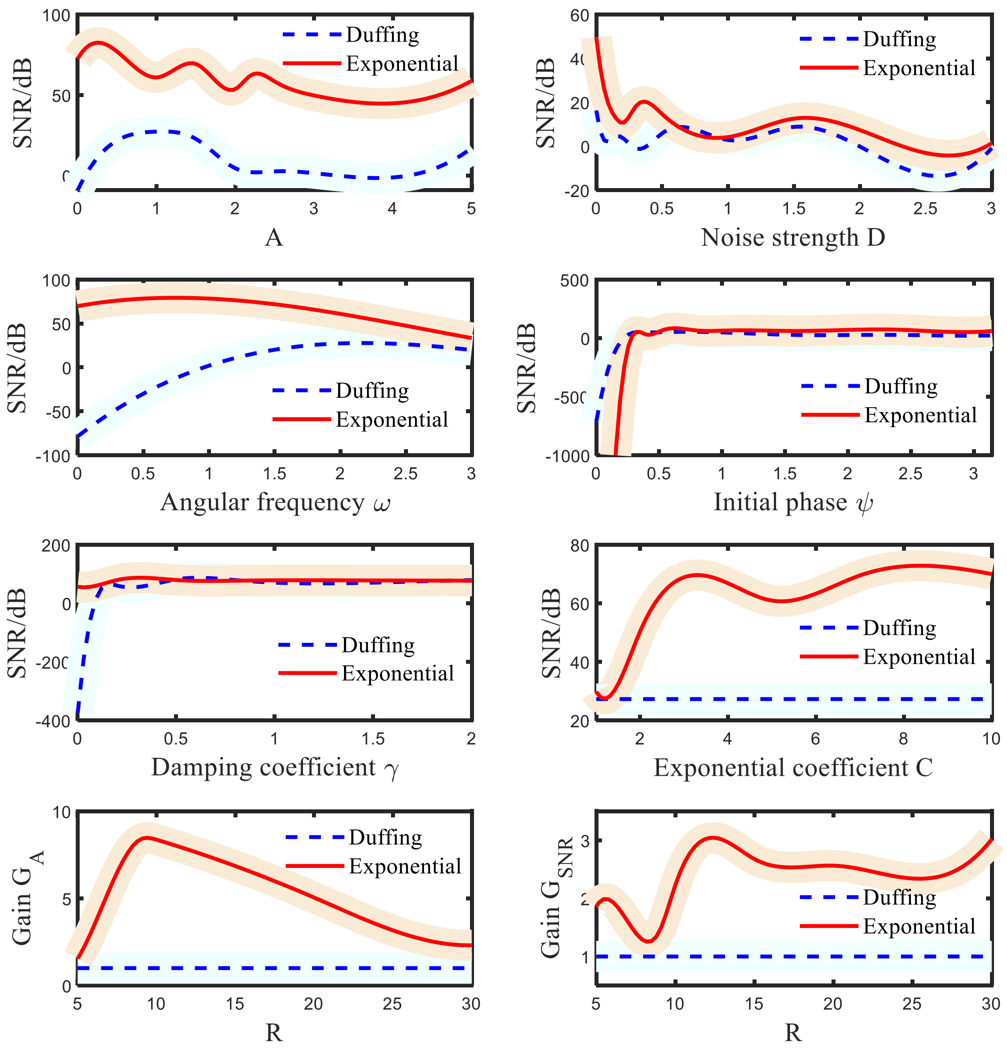

Figure S2. Relationships between the chaotic system parameters and output signal SNR amd

gain coefficients.

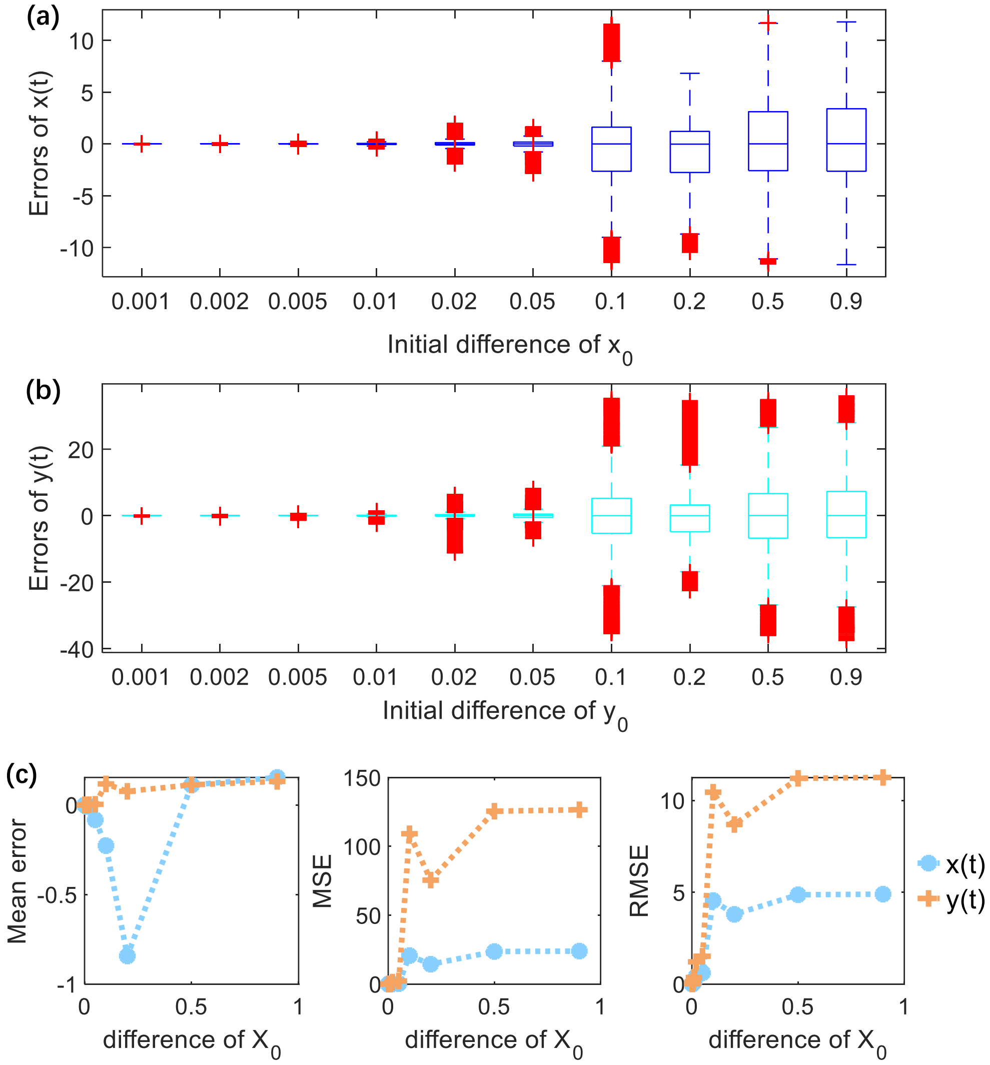

Figure S3 Output errors and performance evaluation for Duffing model. a) Error of output x(t) when the initial input difference of x_0 changes. b) Error of output y(t) when the initial input difference of y_0 changes. c) Change trends of output errors for x(t) and y(t) when the initial difference of x_0 varies within the values of [0.001, 0.002, 0.005, 0.01, 0.02, 0.05, 0.1, 0.2, 0.5, 0.9].

Supplementary Note 2. Lyapunov exponent

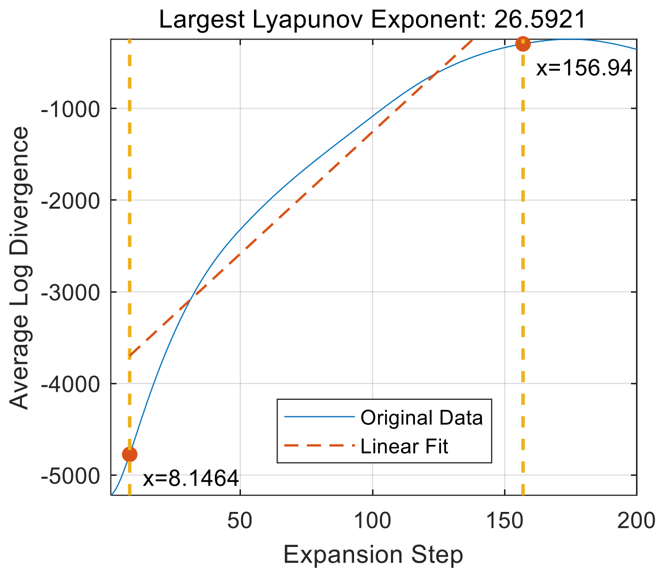

Figure S4. Calculation results for the largest Lyapunov exponent.

Table S3. Lyapunov exponents under different parameters of embedded dimension (Dim), ending time (T), and expansion range(eR).

Dim	1	2	3	4	5	6	7	8	9	10
Ly	0.7311	1.2059	1.1010	1.0006	0.9145	0.8528	0.8017	0.7438	0.6854	0.6230
T	1	5	10	20	30	50	100	200	500	1000
Ly	1.2833	1.1974	1.1010	0.8862	0.7449	0.4942	0.1278	0.0248	0.1659	0.2742
fs	100	200	300	500	600	700	800	1000	1500	2000
Ly	1.1010	2.2019	3.3029	5.5049	6.6058	7.7068	8.8078	11.0097	16.5146	22.0194
eR	10	50	100	150	200	250	300	500	800	1000
Ly	3.3341	3.0295	1.9184	1.4733	1.1010	0.7746	0.5367	0.2281	0.0979	0.0722

Supplementary Note 3. Entropy analysis

a) Topological entropy

Table S4. Topological entropy (TE) for the output of the natural exponential three-dimensional (3D) chaotic system under different system parameters.

(a) TE for the output of the natural exponential 3D chaotic system under different system parameters of *a*.

a	-5	-1	-0.5	-0.3	-0.2	-0.1	-0.01	0	0.01	0.1
x(t)	0.9327	0.9372	0.9353	0.9362	0.9369	0.9347	0.9359	0.9368	0.9359	0.9358
y(t)	0.9359	0.9368	0.9366	0.9353	0.9345	0.9374	0.9372	0.9375	0.9379	0.9383
z(t)	0.9402	0.9517	0.9517	0.9529	0.9492	0.9543	0.9515	0.9523	0.9555	0.9617

(b) TE results for the output of the natural exponential 3D chaotic system under different system parameters of b.

b	0.1	0.2	0.3	0.5	1	2	3	5	7	10
x(t)	0.9373	0.9377	0.9366	0.9383	0.9372	0.9365	0.9348	0.9370	0.9367	0.9402
y(t)	0.9379	0.9376	0.9381	0.9396	0.9399	0.9288	0.9388	0.9451	0.9427	0.9503

z(t)	0.9864	0.9786	0.9853	0.9708	0.9737	0.9678	0.9616	0.9656	0.9602	0.9649

(c) TE results for the output of the natural exponential 3D chaotic system under different system parameters of c.

c	-10	-5	-1	0	1	5	10	20	30	50
x(t)	0.9369	0.9356	0.9354	0.9343	0.9362	0.9348	0.9366	0.9377	0.9368	0.9357
y(t)	0.9372	0.9366	0.9356	0.9294	0.9353	0.9367	0.9393	0.9359	0.9393	0.9401
z(t)	0.9564	0.9622	0.9539	0.9426	0.9529	0.9601	0.9681	0.9753	0.9842	0.9991

(d) TE results for the output of the natural exponential 3D chaotic system under different system parameters of d.

d	-7	-5	-1	0	1	3	5	15	20	50
x(t)	0.9335	0.9315	0.9373	0.9326	0.9373	0.9373	0.9315	0.9337	0.9354	0.9332
y(t)	0.9410	0.9396	0.9379	0.9351	0.9379	0.9387	0.9396	0.9447	0.9369	0.9455
z(t)	1.0132	1.0021	0.9864	0.9872	0.9864	1.0011	1.0021	0.9548	0.9476	0.9537

(e) TE results of x(t) for chordal graph.

TE	A	B	C	D	E	F	G	H	I	J
xa	0.9327	0.9372	0.9353	0.9362	0.9369	0.9347	0.9359	0.9368	0.9359	0.9358
xb	0.9373	0.9377	0.9366	0.9383	0.9372	0.9365	0.9348	0.9370	0.9367	0.9402
xc	0.9369	0.9356	0.9354	0.9343	0.9362	0.9348	0.9366	0.9377	0.9368	0.9357
xd	0.9335	0.9315	0.9373	0.9326	0.9373	0.9373	0.9315	0.9337	0.9354	0.9332

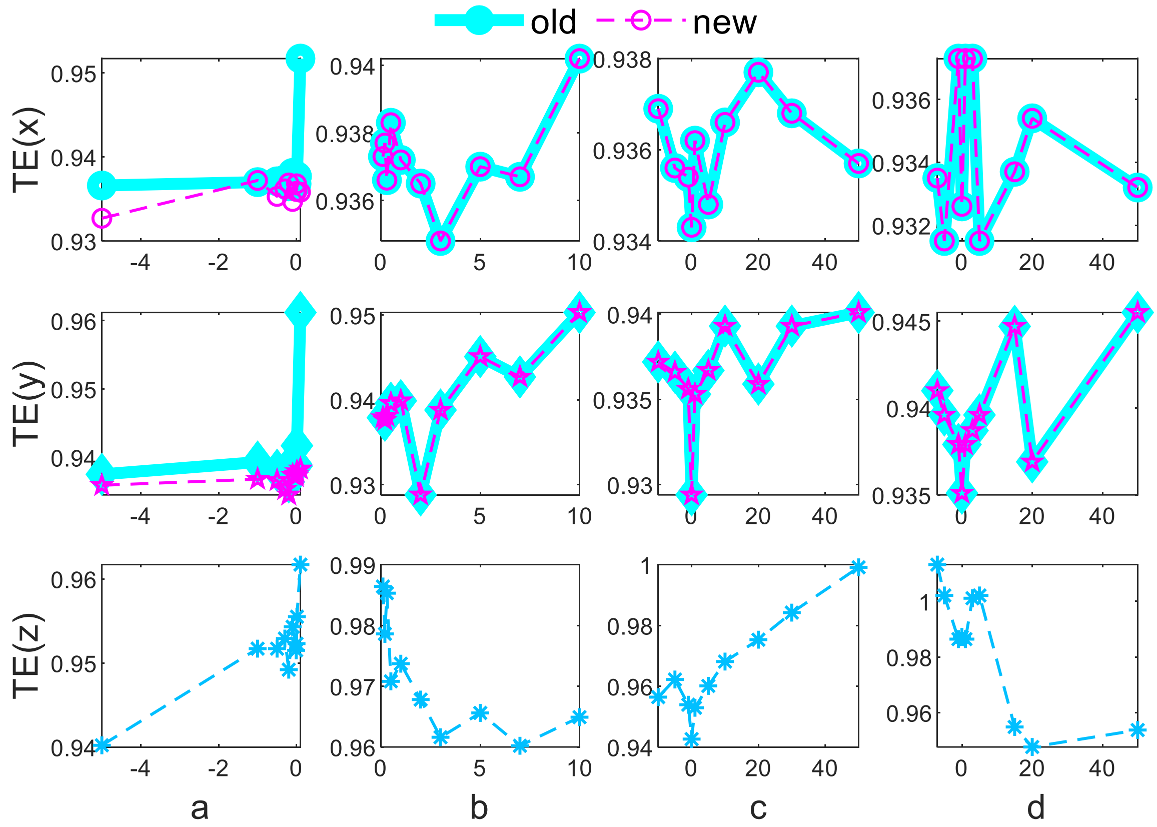

Figure S5. TE of x(t) (upper part, TE(x)), y(t) (middle part, TE(y)) and z(t) (lower part, TE(z)). The system parameters of *a*, b, c and d varies within the scope of [-5, -1, -0.5, -0.3, -0.2, -0.1, -0.01, 0, 0.01, 0.1], [0.1, 0.2, 0.3, 0.5, 1, 2, 3, 5, 7, 10], [-10, -5, -1, 0, 1, 5, 10, 20, 30, 50] and [-7, -5, -1, 0, 1, 3, 5, 15, 20, 50], respectively.

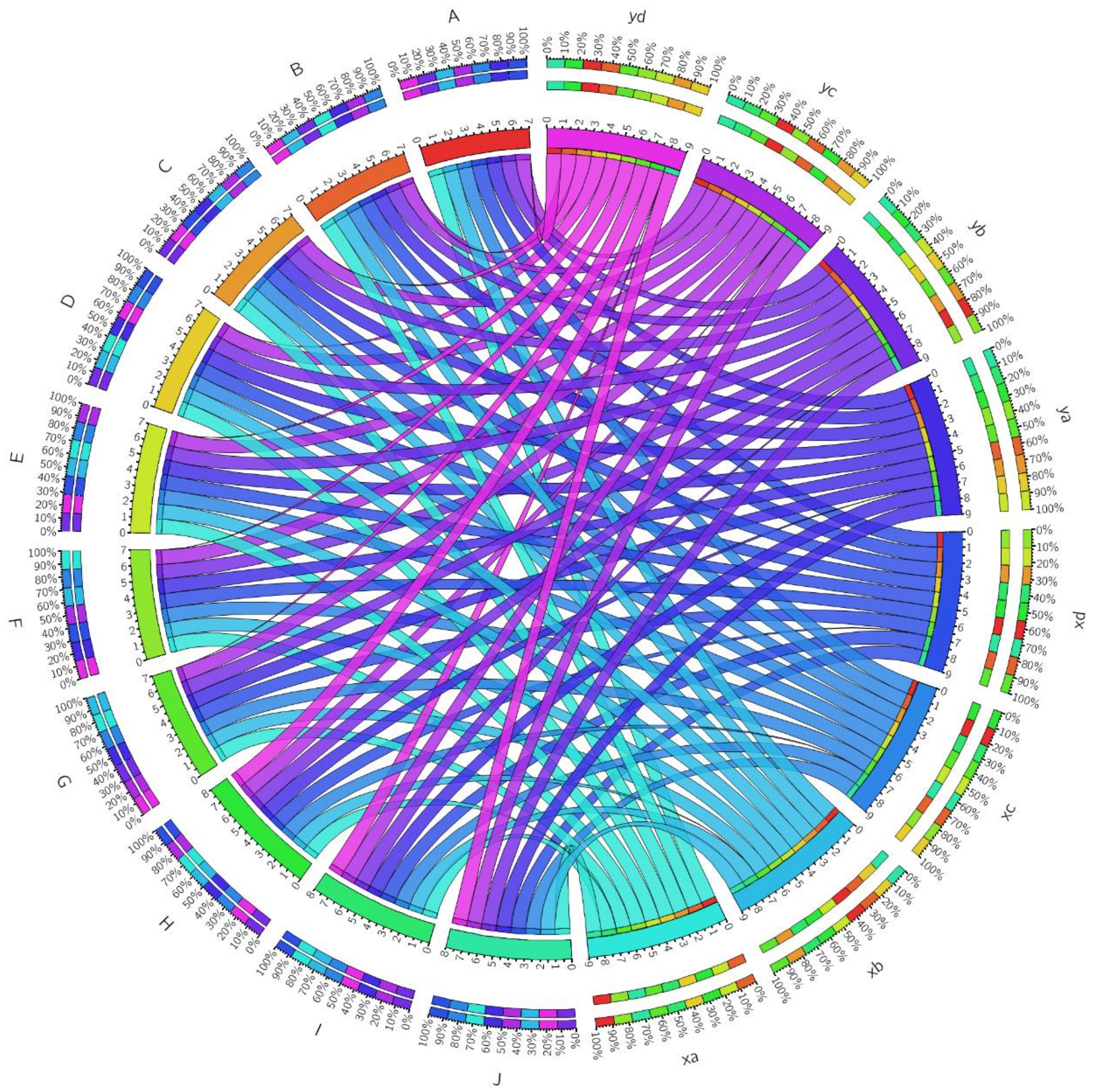

Figure S6. Chordal graph for TE in x-y plane. xa, xb, xc and xd express the corresponding TE of x(t), while ya, yb, yc and yd mean the TE for y(t) when the system parameters of *a*, b, c and d varies within the scope of [-5, -1, -0.5, -0.3, -0.2, -0.1, -0.01, 0, 0.01, 0.1], [0.1, 0.2, 0.3, 0.5, 1, 2, 3, 5, 7, 10], [-10, -5, -1, 0, 1, 5, 10, 20, 30, 50] and [-7, -5, -1, 0, 1, 3, 5, 15, 20, 50], respectively. A to J represents ten groups of system parameters.

b) Approximate entropy

Table S5. Approximate entropy (AE) for the output of the natural exponential 3D chaotic system under different system parameters.

(a) AE for the output of the natural exponential 3D chaotic system under different system parameters of *a*.

a	-5	-1	-0.5	-0.3	-0.2	-0.1	-0.01	0	0.01	0.1
x(t)	0.0721	0.1184	0.1003	0.1278	0.1541	0.1427	0.1312	0.1060	0.1715	0.0446

y(t)	0.0716	0.0406	0.0331	0.0176	0.0162	0.0030	0.0126	0.0717	0.0010	-0.0010
z(t)	0.0045	0.0043	0.0048	0.0044	0.0048	0.0042	0.0048	0.0047	0.0078	0.0797

(b) AE for the output of the natural exponential 3D chaotic system under different system parameters of b.

b	0.1	0.2	0.3	0.5	1	2	3	5	7	10
x(t)	0.1278	0.1725	0.1577	0.1602	0.1416	0.1655	0.1588	0.1448	0.1613	0.1666
y(t)	0.0176	0.0359	0.0201	0.0068	0.0266	0.0317	0.0498	0.0375	0.0430	0.0372
z(t)	0.0044	0.0043	0.0043	0.0045	0.0039	0.0043	0.0040	0.0039	0.0044	0.0040

(c) AE for the output of the natural exponential 3D chaotic system under different system parameters of c.

c	-10	-5	-1	0	1	5	10	20	30	50
x(t)	0.1261	0.0897	0.0927	0	0.0693	0.0869	0.1076	0.1218	0.1496	0.1724
y(t)	0.0707	0.0945	0.1134	0	0.1035	0.0751	0.0912	0.0665	0.0379	0.0157
z(t)	0.0047	0.0038	0.0030	-0.001	0.0039	0.0045	0.0039	0.0050	0.0042	0.0041

(d) AE for the output of the natural exponential 3D chaotic system under different system parameters of d.

d	-7	-5	-1	0	1	3	5	15	20	50
x(t)	0.0605	0.0814	0.1278	0.0386	0.1278	0.0848	0.0814	0.0849	0.0760	0.0543
y(t)	0.0253	0.0376	0.0176	0.1781	0.0176	0.0353	0.0376	0.0959	0.0959	0.0864
z(t)	0.0351	0.0506	0.0044	-0.0010	0.0044	0.0180	0.0506	0.0775	0.0872	-0.0005

(e) AE for the output of the natural exponential 3D chaotic system under different system parameters of L.

L	2	4	6	8	10	12	14	16	18	20
x(t)	0.3984	0.1611	0.1101	0.0927	0.0855	0.0692	0.0571	0.0472	0.0449	0.0290
y(t)	0.1516	0.0388	0.0142	0.0107	0.0049	0.0030	0.0010	-0.0010	-0.0010	-0.0010
z(t)	0.0043	0.0044	0.0044	0.0044	0.0045	0.0045	0.0045	0.0046	0.0046	0.0047

(f) AE for the output of the natural exponential 3D chaotic system under different system parameters of r.

r	0.01	0.1	0.2	0.3	0.4	0.5	0.6	0.7	0.8	0.9
x(t)	-0.0010	0.0387	0.0979	0.1122	0.1128	0.1278	0.1236	0.1273	0.1298	0.1352
y(t)	-0.0010	-0.0010	0.0010	0.0087	0.0106	0.0176	0.0370	0.0425	0.0437	0.0592
z(t)	0.0044	0.0044	0.0044	0.0044	0.0044	0.0044	0.0044	0.0044	0.0044	0.0044

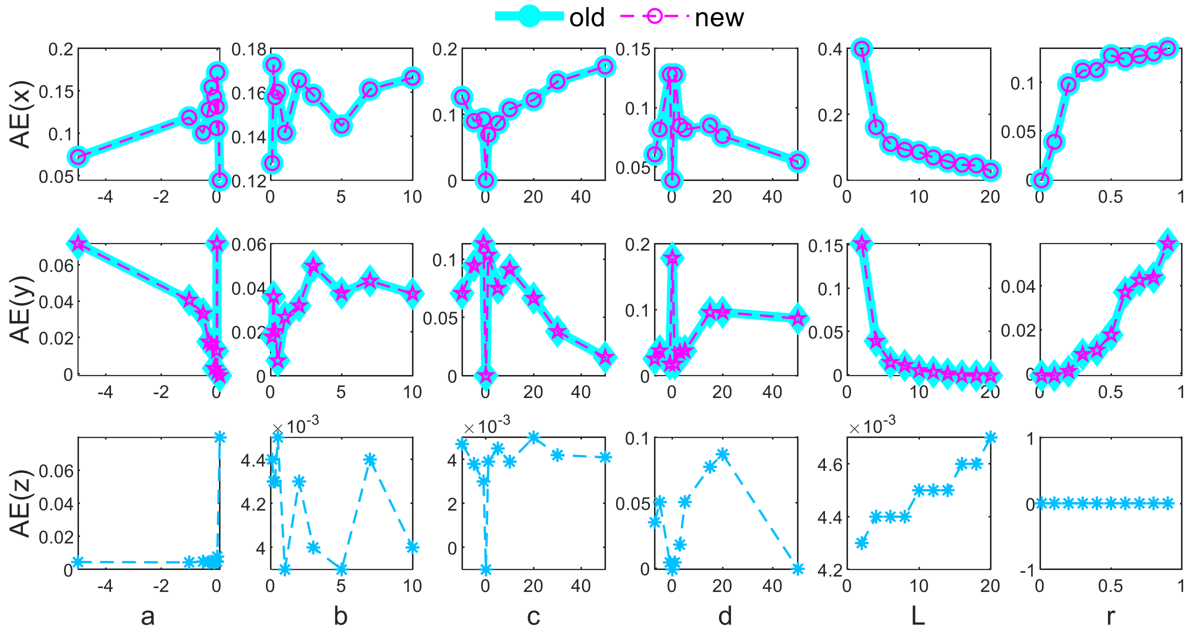

Figure S7. AE of x(t) (upper part, AE(x)), y(t) (middle part, AE(y)) and z(t) (lower part, AE(z)). The system parameters of *a*, b, c, d, L and r varies within the scope of [-5, -1, -0.5, -0.3, -0.2, -0.1, -0.01, 0, 0.01, 0.1], [0.1, 0.2, 0.3, 0.5, 1, 2, 3, 5, 7, 10], [-10, -5, -1, 0, 1, 5, 10, 20, 30, 50], [-7, -5, -1, 0, 1, 3, 5, 15, 20, 50], [2, 4, 6, 8, 10, 12, 14, 16, 18, 20] and [0.01, 0.1, 0.2, 0.3, 0.4, 0.5, 0.6, 0.7, 0.8, 0.9], respectively.

c) Shannon entropy

Table S6. Shannon entropy (SE) for the output of the natural exponential 3D chaotic system under different system parameters.

(a) SE for the output of the natural exponential 3D chaotic system under different system parameters of *a*.

a	-5	-1	-0.5	-0.3	-0.2	-0.1	-0.01	0	0.01	0.1
x(t)	2.2200	2.6282	2.8433	2.7460	2.9508	3.0185	2.8009	2.7513	3.1259	3.6712
y(t)	2.4368	3.2474	3.4043	3.4278	3.4932	3.5715	3.2132	3.1999	3.5979	3.4108
z(t)	0.7268	0.6919	0.4162	0.4254	0.5458	0.3642	0.2358	0.8197	0.3186	0.4466

(b) SE for the output of the natural exponential 3D chaotic system under different system parameters of b.

b	0.1	0.2	0.3	0.5	1	2	3	5	7	10
x(t)	2.7460	2.7795	2.9317	3.0424	2.8111	2.6832	2.9007	2.8242	3.1130	3.1127
y(t)	3.4278	3.5322	3.6631	3.6560	3.6554	3.7523	3.8658	3.9279	3.9739	4.0314
z(t)	0.4254	0.5081	0.5370	0.5711	0.6101	0.7864	0.8295	1.0928	1.1228	1.1003

(c) SE for the output of the natural exponential 3D chaotic system under different system parameters of c.

c	-10	-5	-1	0	1	5	10	20	30	50
x(t)	2.8148	2.6015	2.4199	2.5341	2.2295	2.8152	2.7224	2.7995	2.6450	2.6447

y(t)	3.1597	2.9209	2.5109	2.6155	2.5881	3.1690	3.1925	3.2975	3.4154	3.4859
z(t)	1.2027	1.3023	0.9874	2.4776	0.1443	0.5656	0.4971	0.4492	0.3765	0.4590

(d) SE for the output of the natural exponential 3D chaotic system under different system parameters of d.

d	-7	-5	-1	0	1	3	5	15	20	50
x(t)	3.4046	3.0101	2.7460	3.6535	2.7460	3.0544	3.0101	3.5657	3.4731	4.4446
y(t)	2.4327	3.4070	3.4278	3.6337	3.4278	3.1856	3.4070	3.4828	3.3019	3.7397
z(t)	1.9923	1.6910	0.4254	2.4777	0.4254	1.5353	1.6910	2.4432	2.4441	2.4742

(e) SE for the output of the natural exponential 3D chaotic system under different system parameters of w.

w	1	2	3	4	5	6	7	8	9	10
x(t)	2.1550	2.4534	2.7460	3.0321	3.3106	3.5801	3.8385	4.0680	4.2791	4.4636
y(t)	2.2322	2.8712	3.4278	3.8900	4.2473	4.5420	4.7857	4.9970	5.1867	5.3488
z(t)	0.4003	0.4156	0.4254	0.4352	0.4449	0.4546	0.4644	0.4740	0.4837	0.4933

(f) SE for the output of the natural exponential 3D chaotic system under different system parameters of q.

q	2	4	6	8	10	12	14	16	18	20
x(t)	0.7270	1.4835	2.0447	2.4514	2.7460	3.0336	3.3289	3.4833	3.7044	3.8500
y(t)	0.9338	1.9406	2.5698	3.0901	3.4278	3.7717	3.9959	4.2010	4.3699	4.5523
z(t)	0.3077	0.3642	0.3877	0.4111	0.4254	0.4345	0.4345	0.4487	0.4578	0.4720

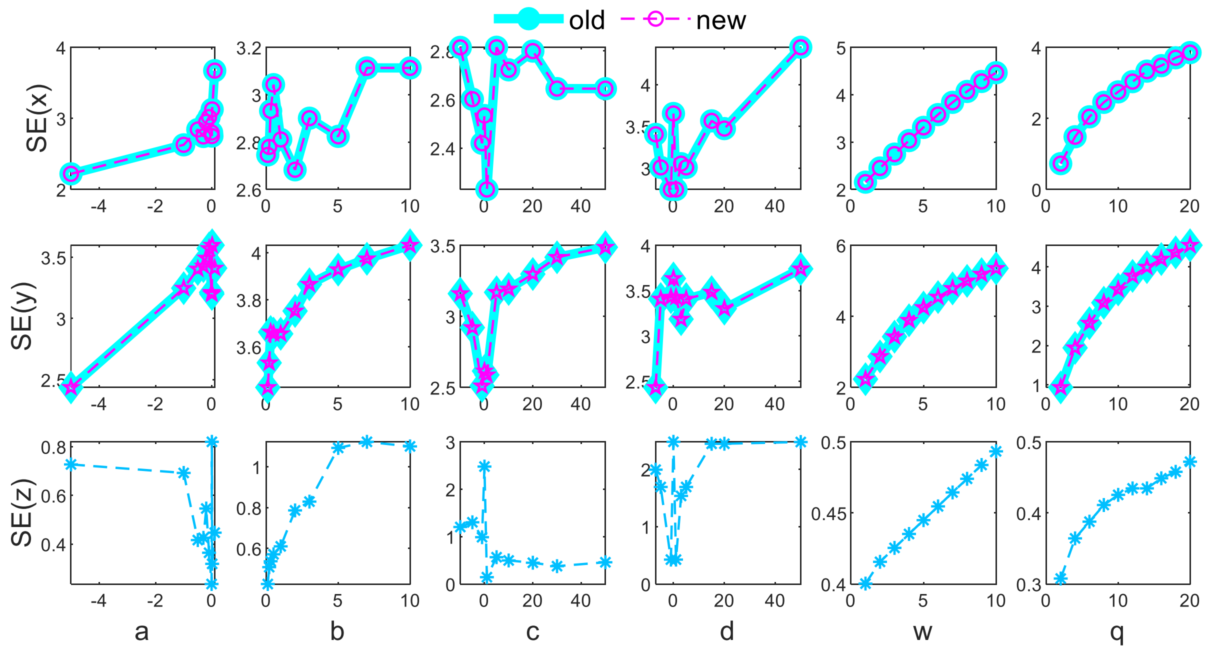

Figure S8. SE of x(t) (upper part, SE(x)), y(t) (middle part, SE(y)) and z(t) (lower part, SE(z)). The system parameters of *a*, b, c, d, w and q varies within the scope of [-5, -1, -0.5, -0.3, -0.2, -0.1, -0.01, 0, 0.01, 0.1], [0.1, 0.2, 0.3, 0.5, 1, 2, 3, 5, 7, 10], [-10, -5, -1, 0, 1, 5, 10, 20, 30, 50], [-7, -5, -1, 0, 1, 3, 5, 15, 20, 50], [1, 2, 3, 4, 5, 6, 7, 8, 9, 10] and [2, 4, 6, 8, 10, 12, 14, 16, 18, 20], respectively.

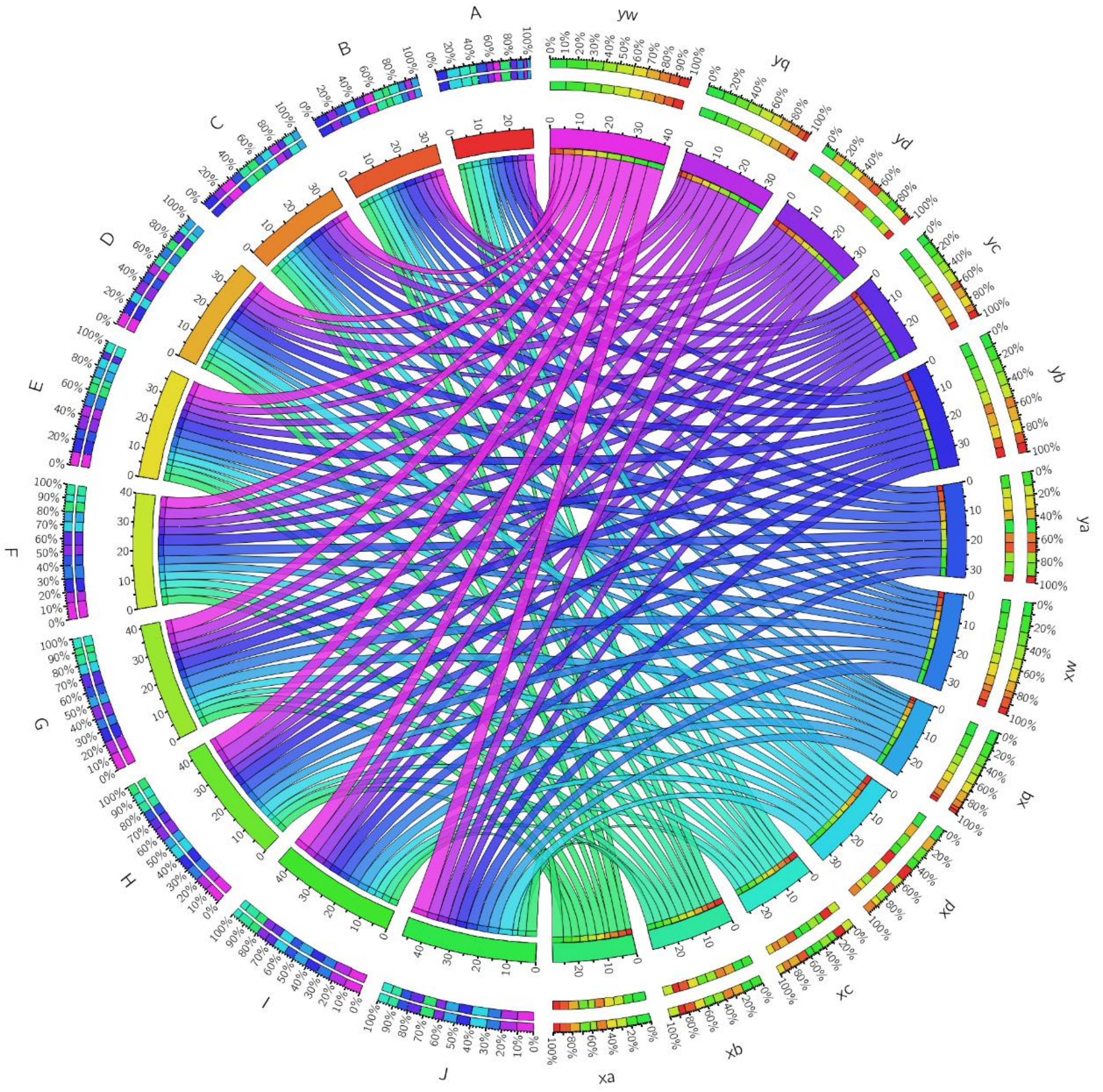

Figure S9. Chordal graph for SE in x-y plane. xa, xb, xc xd, xq and xw express the corresponding SE of x(t), while ya, yb, yc, yd, yq and yw mean the SE for y(t) when the system parameters of *a*, b, c, d, q and w varies within the scope of [-5, -1, -0.5, -0.3, -0.2, -0.1, -0.01, 0, 0.01, 0.1], [0.1, 0.2, 0.3, 0.5, 1, 2, 3, 5, 7, 10], [-10, -5, -1, 0, 1, 5, 10, 20, 30, 50], [-7, -5, -1, 0, 1, 3, 5, 15, 20, 50], [2, 4, 6, 8, 10, 12, 14, 16, 18, 20] and [1, 2, 3, 4, 5, 6, 7, 8, 9, 10], respectively. A to J represents ten groups of system parameters.

d) Fuzzy entropy

Table S7. Fuzzy entropy (FE) for the output of the natural exponential 3D chaotic system under different system parameters.

(a) FE for the output of the natural exponential 3D chaotic system under different system parameters of *a*.

a	-5	-1	-0.5	-0.3	-0.2	-0.1	-0.01	0	0.01	0.1

x(t)	0.0003	0.0007	0.0014	0.0013	0.0018	0.0026	0.0035	0.0017	0.0054	0.0297
y(t)	0.0040	0.0120	0.0136	0.0132	0.0130	0.0140	0.0142	0.0128	0.0139	0.0343
z(t)	0.0000	0.0002	0.0002	0.0002	0.0001	0.0003	0.0004	0.0000	0.0004	0.0027

(b) FE for the output of the natural exponential 3D chaotic system under different system parameters of b.

b	0.1	0.2	0.3	0.5	1	2	3	5	7	10
x(t)	0.0013	0.0012	0.0022	0.0027	0.0024	0.0027	0.0019	0.0023	0.0038	0.0031
y(t)	0.0132	0.0156	0.0202	0.0246	0.0308	0.0358	0.0413	0.0481	0.0560	0.0621
z(t)	0.0002	0.0002	0.0002	0.0001	0.0001	0.0001	0.0001	0.0001	0.0000	0.0000

(c) FE for the output of the natural exponential 3D chaotic system under different system parameters of c.

c	-10	-5	-1	0	1	5	10	20	30	50
x(t)	0.0009	0.0008	0.0004	0.0002	0.0005	0.0011	0.0008	0.0011	0.0008	0.0011
y(t)	0.0059	0.0040	0.0013	0.0003	0.0014	0.0038	0.0059	0.0091	0.0109	0.0157
z(t)	0.0003	0.0003	0.0002	-0.0000	0.0002	0.0002	0.0002	0.0001	0.0002	0.0002

(d) FE for the output of the natural exponential 3D chaotic system under different system parameters of d.

d	-7	-5	-1	0	1	3	5	15	20	50
x(t)	0.0069	0.0077	0.0013	0.0203	0.0013	0.0031	0.0077	0.0286	0.0501	0.2073
y(t)	0.0104	0.0139	0.0132	0.0204	0.0132	0.0106	0.0139	0.0288	0.0497	0.2219
z(t)	0.0002	0.0002	0.0002	0.0000	0.0002	0.0002	0.0002	0.0001	0.0000	-0.0000

(e) FE for the output of the natural exponential 3D chaotic system under different system parameters of dim.

dim	2	4	6	8	10	12	14	16	18	20
x(t)	0.0008	0.0019	0.0030	0.0042	0.0052	0.0062	0.0071	0.0078	0.0083	0.0086
y(t)	0.0079	0.0185	0.0280	0.0354	0.0407	0.0441	0.0460	0.0467	0.0463	0.0449
z(t)	0.0001	0.0003	0.0004	0.0006	0.0007	0.0007	0.0008	0.0008	0.0008	0.0008

(f) FE for the output of the natural exponential 3D chaotic system under different system parameters of wid.

wid	2	4	6	8	10	12	14	16	18	20
x(t)	0.0013	0.0007	0.0004	0.0003	0.0003	0.0002	0.0002	0.0002	0.0001	0.0001
y(t)	0.0132	0.0067	0.0045	0.0034	0.0027	0.0023	0.0019	0.0017	0.0015	0.0014
z(t)	0.0002	0.0001	0.0001	0	0	0	0	0	0	0

(g) FE for the output of the natural exponential 3D chaotic system under different system parameters of st.

st	1	2	3	4	5	6	7	8	9	10
x(t)	0.0095	0.0013	0.0002	0	0	0	0	0	0	0
y(t)	0.0293	0.0132	0.0058	0.0027	0.0013	0.0007	0.0004	0.0002	0.0001	0.0001
z(t)	0.0011	0.0002	0	0	0	0	0	0	0	0

e) Conditional entropy

Table S8. Conditional entropy (CE) for the output of the natural exponential 3D chaotic system under different system parameters.

(a) CE for the output of the natural exponential 3D chaotic system under different system parameters of *a*.

a	-5	-1	-0.5	-0.3	-0.2	-0.1	-0.01	0	0.01	0.1
x(t)	0.0202	0.0201	0.0259	0.0259	0.0259	0.0201	0.0362	0.0259	0.0776	0.1875
y(t)	0.0777	0.1236	0.1373	0.1306	0.1340	0.1374	0.1306	0.1200	0.1306	0.1875
z(t)	0.0072	0.0072	0.0072	0.0072	0.0072	0.0072	0.0072	0.0072	0.0072	0.0000

(b) CE for the output of the natural exponential 3D chaotic system under different system parameters of b.

b	0.1	0.2	0.3	0.5	1	2	3	5	7	10
x(t)	0.0259	0.0256	0.0258	0.0202	0.0200	0.0257	0.0256	0.0255	0.0202	0.0256
y(t)	0.1306	0.1474	0.1507	0.1633	0.1782	0.1961	0.2044	0.2182	0.2338	0.2338
z(t)	0.0072	0.0072	0.0072	0.0072	0.0072	0.0072	0.0072	0.0072	0.0072	0.0072

(c) CE for the output of the natural exponential 3D chaotic system under different system parameters of c.

c	-10	-5	-1	0	1	5	10	20	30	50
x(t)	0.0203	0.0203	0.0201	0.0260	0.0140	0.0202	0.0200	0.0260	0.0256	0.0256
y(t)	0.1016	0.0776	0.0467	0.0314	0.0601	0.0819	0.0975	0.1128	0.1271	0.1474
z(t)	0.0067	0.0068	0.0067	0.0072	0.0072	0.0072	0.0072	0.0072	0.0072	0.0072

(d) CE for the output of the natural exponential 3D chaotic system under different system parameters of d.

d	-7	-5	-1	0	1	3	5	15	20	50
x(t)	0.1054	0.0798	0.0259	0.1665	0.0259	0.0559	0.0798	0.1904	0.2338	0.4081
y(t)	0.1054	0.1408	0.1306	0.1665	0.1306	0.1271	0.1408	0.1904	0.2312	0.4082
z(t)	0.0072	0.0071	0.0072	0.0072	0.0072	0.0072	0.0071	0.0072	0.0072	0.0072

(e) CE for the output of the natural exponential 3D chaotic system under different system parameters of L (embedded dimension).

L	2	4	6	8	10	12	14	16	18	20
x(t)	0.0259	0.0259	0.0259	0.0260	0.0260	0.0261	0.0205	0.0205	0.0206	0.0206
y(t)	0.1312	0.1299	0.1284	0.1267	0.1249	0.1229	0.1206	0.1180	0.1151	0.1117
z(t)	0.0072	0.0072	0.0073	0.0073	0.0073	0.0073	0.0074	0.0074	0.0074	0.0074

(f) CE for the output of the natural exponential 3D chaotic system under different system parameters of v (number of uniform intervals used in the quantification of the series).

v	2	4	6	8	10	12	14	16	18	20
x(t)	0.0259	0.1249	0.1937	0.2516	0.3154	0.3670	0.4081	0.4291	0.4732	0.5147
y(t)	0.1306	0.2869	0.4114	0.4730	0.5633	0.6288	0.6634	0.7000	0.7436	0.7636
z(t)	0.0072	0.0124	0.0197	0.0208	0.0214	0.0224	0.0240	0.0229	0.0229	0.0247

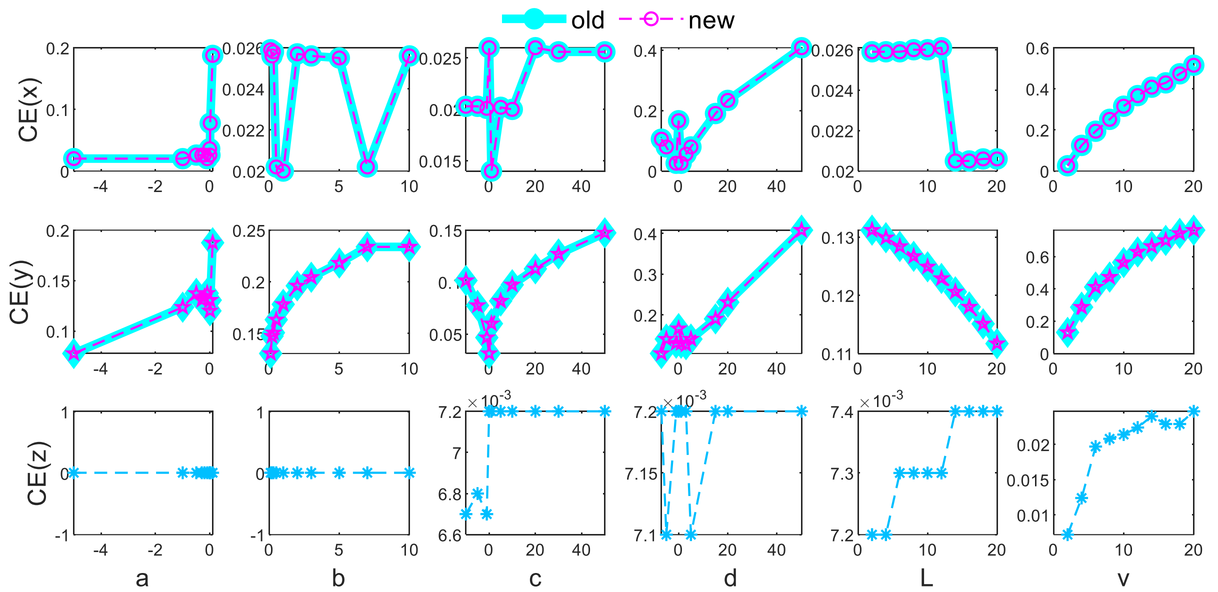

Figure S10. CE of x(t) (upper part, CE(x)), y(t) (middle part, CE(y)) and z(t) (lower part, CE(z)). The system parameters of *a*, b, c, d, L and v varies within the scope of [-5, -1, -0.5, -0.3, -0.2, -0.1, -0.01, 0, 0.01, 0.1], [0.1, 0.2, 0.3, 0.5, 1, 2, 3, 5, 7, 10], [-10, -5, -1, 0, 1, 5, 10, 20, 30, 50], [-7, -5, -1, 0, 1, 3, 5, 15, 20, 50], [2, 4, 6, 8, 10, 12, 14, 16, 18, 20] and [2, 4, 6, 8, 10, 12, 14, 16, 18, 20], respectively.

f) Time series entropy for z(t)

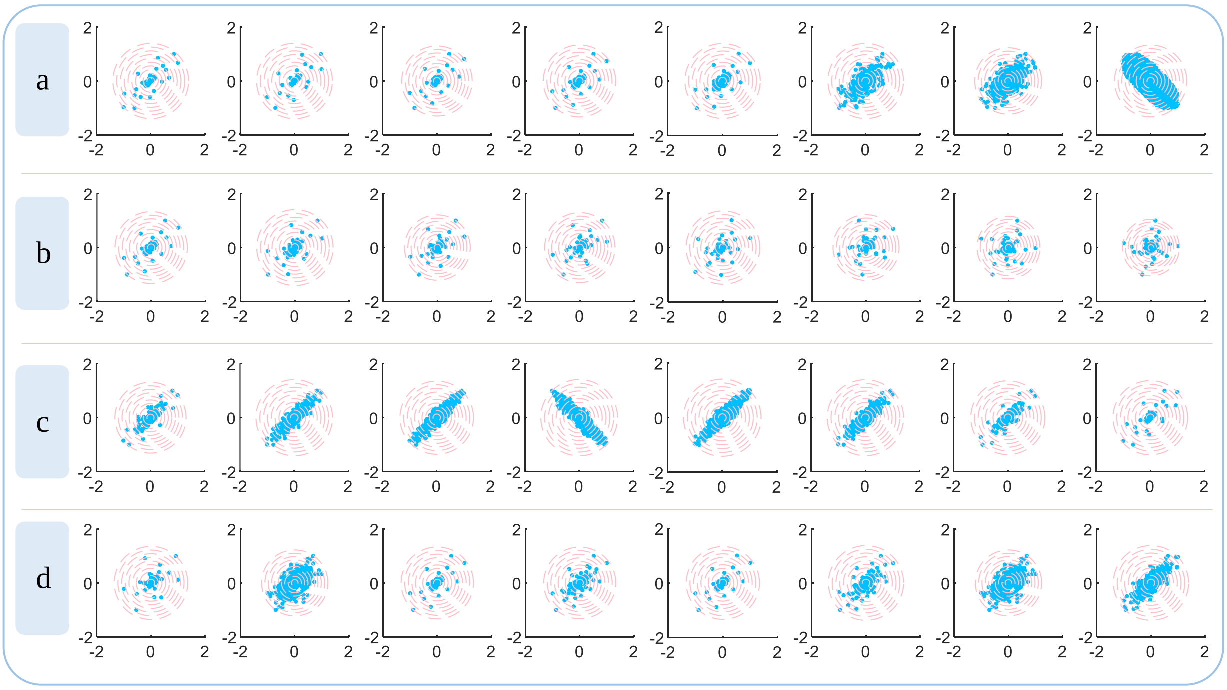

Figure S11. Time series entropy (TSE) measurement for z(t). a-d TSE results for z(t) when the system parameter of a, b, c, and d changes. These parameters are set as *a* = [-5, -1, -0.5, -0.3, -0.1, -0.01, 0.01, 0.1], b = [0.1, 0.2, 0.3, 0.5, 1, 3, 5, 7], c = [-10, -5, -1, 0, 1, 5, 10, 20], d = [-7, -5, -1, 0, 1, 3, 5, 15].

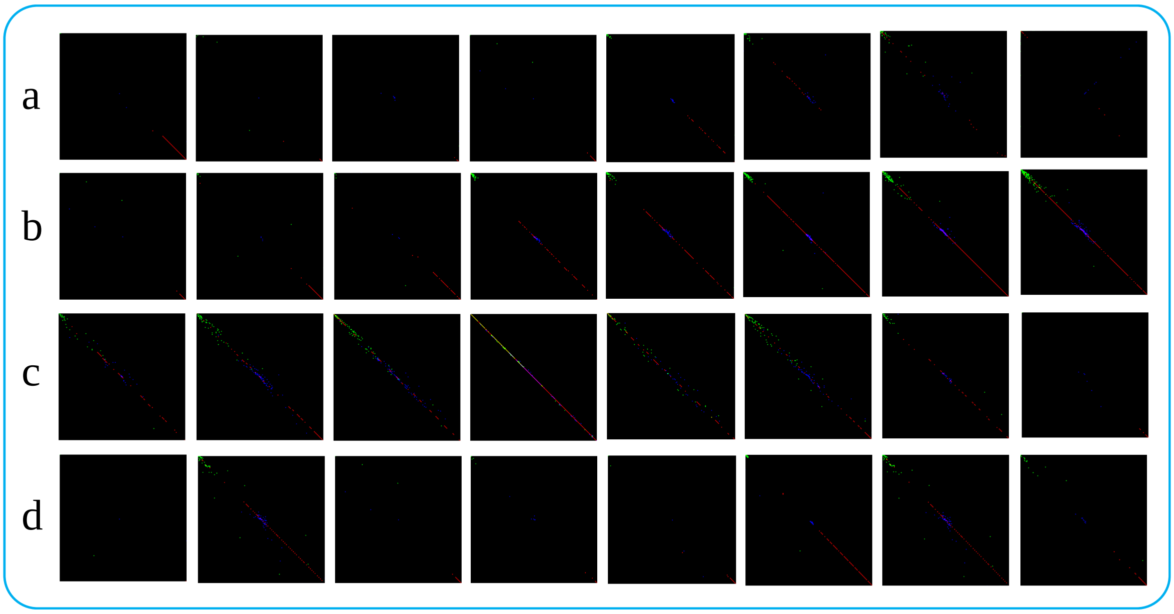

Figure S12. Grid entropy (GE) measurement for z(t). a-d GE for z(t) when the system parameter of a, b, c and d changes as mentioned in **Figure. 8**.

Supplementary Note 4. Time series forecasting for y(t)

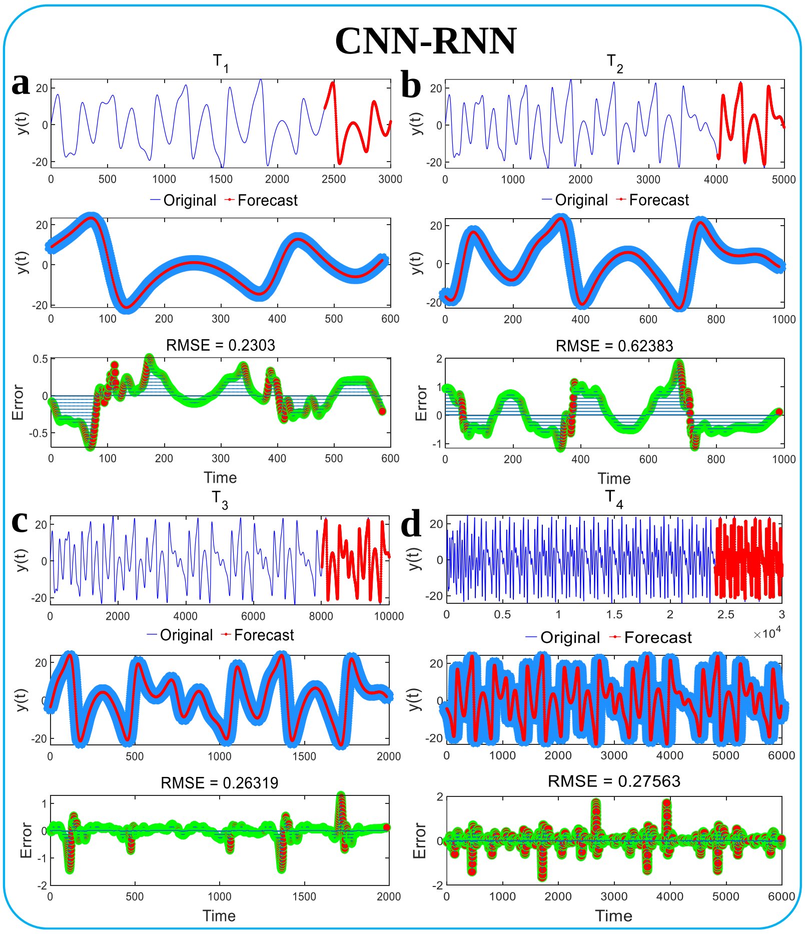

Figure S13. Application of the natural exponential chaotic system in time series prediction

for y(t) by CNN-RNN. a-d Time series prediction results of y(t) through convolutional and recurrent combined neural network (CNN-RNN). The root mean square error (RMSE) for the predicted time series of T_1 to T_4 is 0.2303, 0.62383, 0.26319 and 0.27563, respectively.

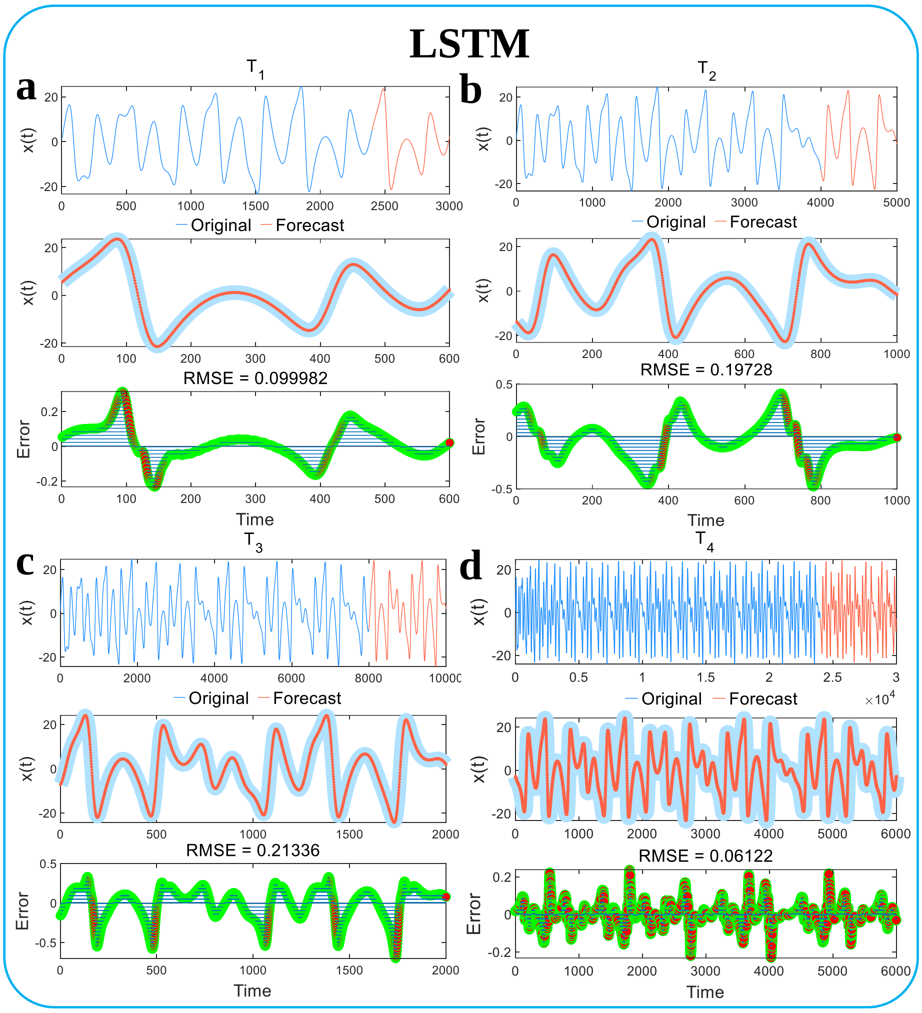

Figure S14. Application of the natural exponential chaotic system in time series prediction for y(t) by LSTM. a-d Time series prediction results of y(t) by long-short term memory neural network (LSTM). The RMSE for the predicted T_1 to T_4 is 0.0999, 0.19728, 0.21336 and 0.06122, respectively.

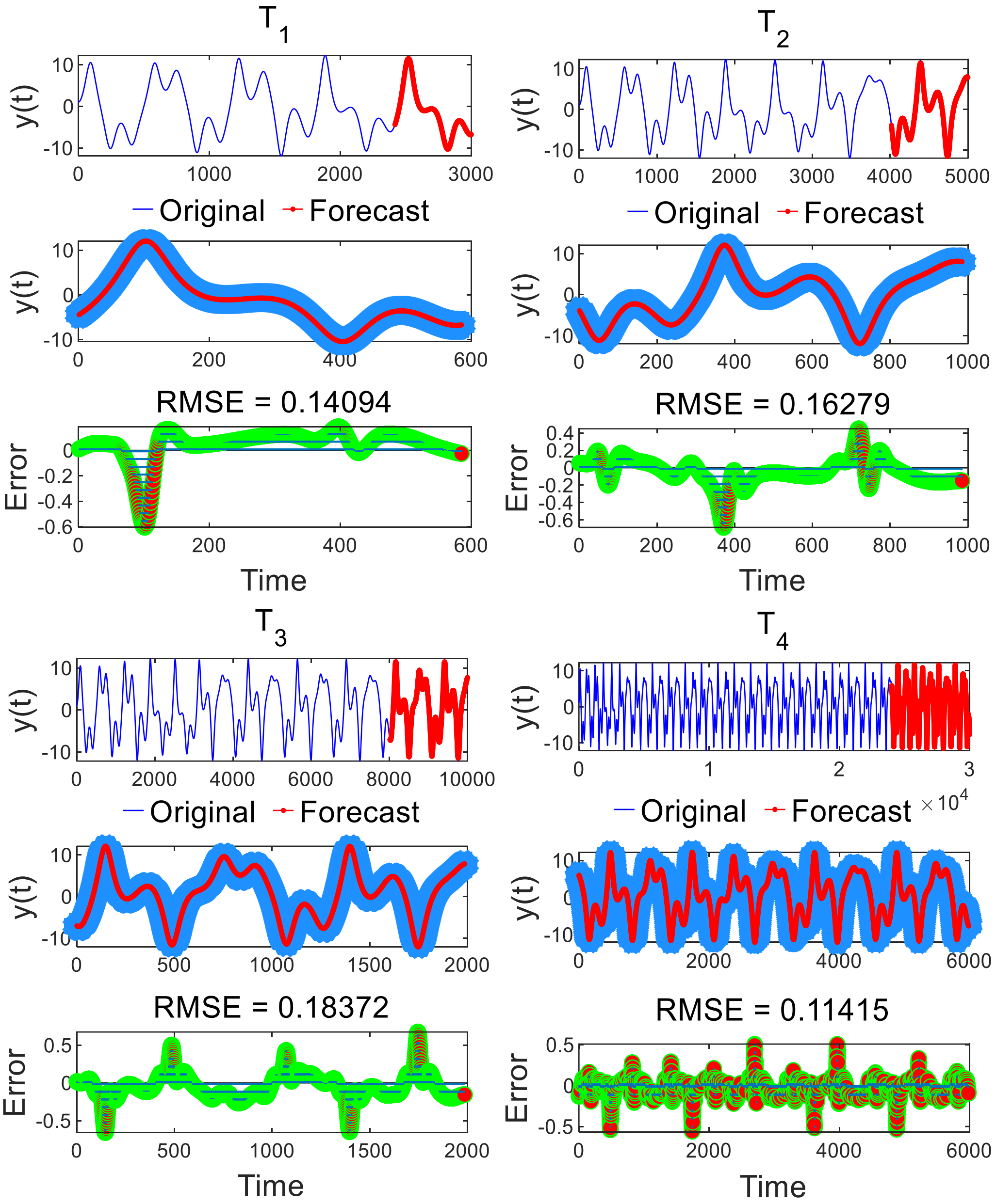

Figure S15. Application and comparison of the RNN in chaotic time series T_1 to T_4 prediction.

Table S9. Horizontal (H), vertical (V) and diagnoal (D) correlation and information entropy (IE) for R-G-B channels of the encrypted image through different chaotic models.

Correlation \ Model		Original	Duffing	Lorenz	Nosehoover	Arneodo	TSUCSI	Aizawa	Rossler	**New**
R	H	0.9774	-0.01354	0.00895	0.01087	-0.00518	-0.02103	0.03922	-0.00305	**-0.00867**
	V	0.98732	0.00099	-0.00869	-0.01092	-0.00554	0.01599	-0.00962	0.00089	**-0.01808**
	D	0.96545	-0.04055	0.01549	-0.01536	0.00299	0.00376	0.00899	0.01473	**-0.00735**
	IE	7.26820	7.99930	7.99930	7.99930	7.99940	7.99940	7.99940	7.99940	**7.99920**
G	H	0.97824	-0.01579	-0.00204	-0.00876	0.01502	-0.00069	-0.01352	-0.00403	**-0.00966**
	V	0.98824	-0.00055	0.00418	0.00246	-0.00495	-0.01113	0.00577	0.00902	**0.00614**
	D	0.96671	-0.00050	-0.01181	0.01317	0.02848	0.00564	-0.01861	-0.01785	**0.00607**
	IE	7.59010	7.99920	7.99940	7.99930	7.99930	7.99940	7.99940	7.99940	**7.99910**
B	H	0.95750	-0.01737	-0.01689	0.01231	0.02113	0.00558	-0.01301	0.00416	**0.00394**
	V	0.97378	0.01203	0.0138	0.01367	0.00940	-0.01287	0.00675	0.01536	**-0.01752**
	D	0.93529	0.00126	0.00594	-0.01247	0.00244	-0.00312	-0.00522	0.00635	**-0.02100**
	IE	6.99510	7.99920	7.99920	7.99940	7.99930	7.99930	7.99930	7.99930	**7.99930**

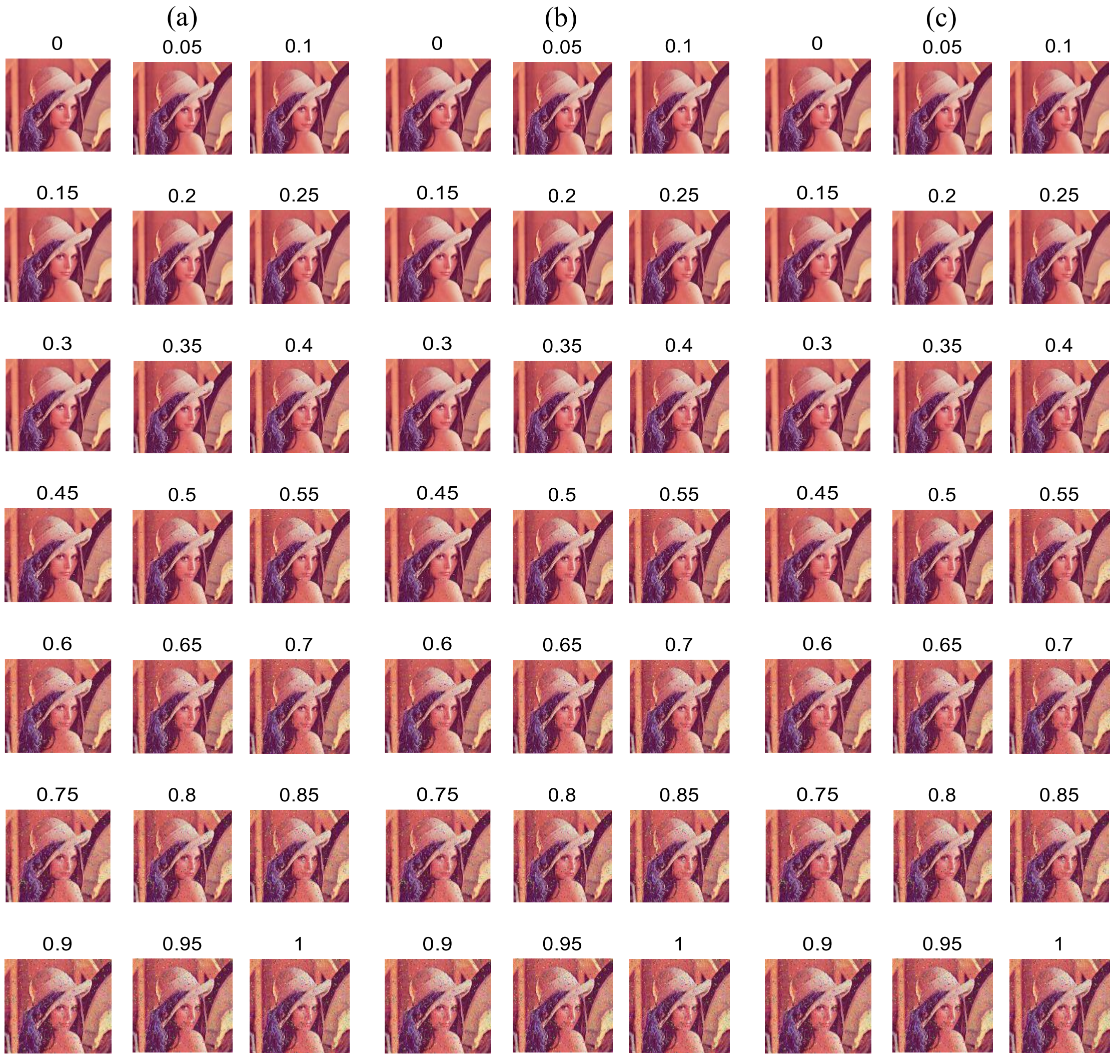
(a)
0
0.05
0.1
0.15
0.2
0.25
0.3
0.35
0.4
0.45
0.5
0.55
0.6
0.65
0.7
0.75
0.8
0.85
0.9
0.95
1
(b)
0
0.05
0.1
0.15
0.2
0.25
0.3
0.35
0.4
0.45
0.5
0.55
0.6
0.65
0.7
0.75
0.8
0.85
0.9
0.95
1
(c)
0
0.05
0.1
0.15
0.2
0.25
0.3
0.35
0.4
0.45
0.5
0.55
0.6
0.65
0.7
0.75
0.8
0.85
0.9
0.95
1

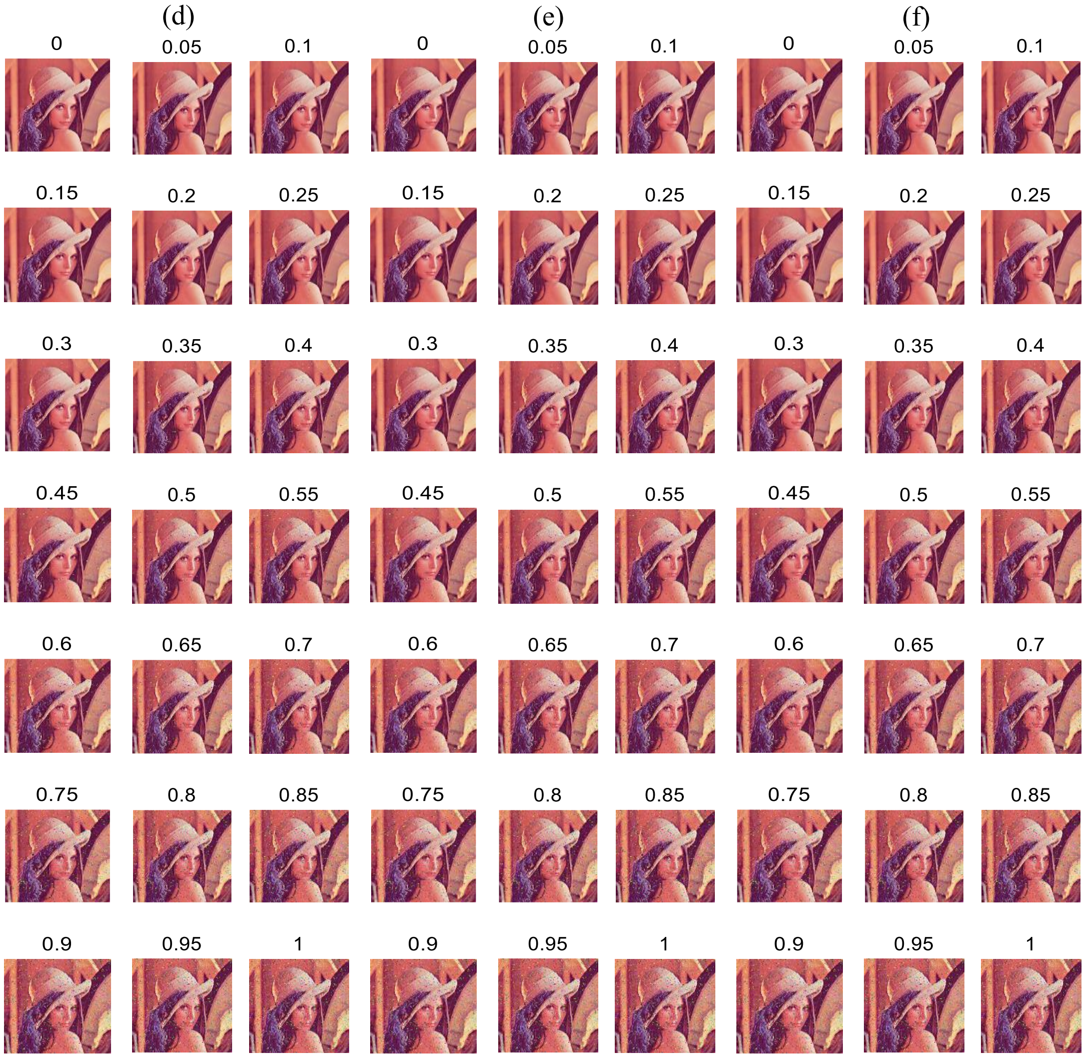

Figure S16. Comparison of the different chaotic system in image encryption and decryption. a) Decrypted image under different noise density by Duffing model. b) Rossler. c) Lorenz. d) NoseHoover. e) Arneodo. f) Aizawa. The labels marked in each image represent the Gaussian noise intensity added to the original image before decrypted.
